# Morphology-adaptive Au-Ag nanowire elastronics for integrated FlexoSERS and bioelectrical sensing

**DOI:** 10.1126/sciadv.aec2162

**Published:** 2026-02-18

**Authors:** Heng Zhang, Yi Chen, Gangsheng Chen, Wuxing Zhang, Cheng Yang, Fan Zhou, Yunqi Zhao, Haoran Deng, Xuan Huang, Yuan An, Guoqun Li, Shuqi Tang, Biao Ma, Wenlong Cheng, Ning Gu

**Affiliations:** ^1^ State Key Laboratory of Digital Medical Engineering, Jiangsu Key Laboratory for Biomaterials and Devices, School of Biological Science and Medical Engineering, Southeast University, Nanjing 211189, China.; ^2^ Southeast University-Monash University Joint Graduate School, Southeast University Suzhou Research Institute, Suzhou 215123, China.; ^3^ School of Biomedical Engineering, Faculty of Engineering, University of Sydney, Darlington, NSW 2008, Australia.; ^4^ Key Laboratory of Quantum Materials and Devices of Ministry of Education, School of Physics, Southeast University, Nanjing 211189, China.; ^5^ Jiangsu Key Laboratory for Cardiovascular Information and Health Engineering Medicine, Nanjing Drum Tower Hospital, Medical School, Nanjing University, Nanjing 210093, China.

## Abstract

We introduce a morphology-adaptive Au-Ag nanowire elastronic platform that conforms to diverse geometries while enabling multimodal optical-electrical sensing. Using a facile yet versatile template-guided growth strategy, vertically aligned Au-Ag nanowire arrays are directly fabricated on 1D nano/microneedles, 2D elastic films, and 3D porous architectures. On 2D substrates, the arrays act as FlexoSERS interfaces with high sensitivity, uniformity (RSD = 7.2%), and durability, maintaining stable SERS signals under 100% strain and after 2500 cycles. On 3D porous sponges, the NWs serve as dry bioelectrical electrodes, enabling stable electrocardiogram (ECG) and electromyogram (EMG) monitoring with long-term stability. Continuous ECG recording, combined with deep learning analysis, enables accurate classification between sleep and wake states. Meanwhile, the EMG signals capture subtle motor activities such as finger bending, typing, and clicking. By uniting strain-tolerant FlexoSERS with reliable bioelectrical sensing across 1D-3D substrates, this platform provides a robust material foundation and a scalable route toward next-generation wearable health monitors, intelligent sleep evaluation, and human-machine interfaces.

## Teaser

A vertically aligned Au-Ag nanowire array as a morphology-adaptive, dual-functional conformal sensing platform.

## INTRODUCTION

Flexible bioelectronic technologies (*1*–*5*) have rapidly advanced as a cornerstone for next-generation health monitoring and medical diagnostics, driven by their promising applications in precision health management (*6*–*8*), personalized disease intervention (*3*, *9*), and intelligent human-machine interfaces (*10*–*12*). Among the various sensing modalities, electrophysiological signals, including electromyographic, electroencephalographic, and electrocardiographic signals, enable real-time monitoring of tissue excitability and physiological rhythms. These signals provide critical insights for early disease diagnosis, pathological mechanism analysis, and neural function rehabilitation and are therefore widely used in wearable and implantable devices (*10*, *13*, *14*). Despite their utility, electrophysiological signals alone reflect only bioelectrical activity and fall short in capturing the full complexity of physiological or pathological processes. In dynamic disease monitoring, particularly for conditions involving complex biochemical changes, the ultrasensitive detection of low-abundance biomarkers in body fluids is equally indispensable (*15*, *16*). These limitations have catalyzed a shift toward multimodal bioelectronic platforms that integrate electrical and chemical sensing within a single device (*17*, *18*), enabling a more holistic and dynamic assessment of physiological states. This paradigm shift imposes new demands on materials design, moving beyond single-signal responsiveness toward integrated, multifunctional architectures capable of operating across diverse physiological environments.

Among the diverse molecular recognition strategies, surface-enhanced Raman scattering (SERS) has emerged as a powerful tool for the analysis of trace biofluids such as sweat, saliva, tears, and interstitial fluid, owing to its label-free operation, high molecular specificity, and exceptional detection sensitivity (*16*, *19*, *20*). By incorporating plasmonic nanostructures (*21*–*24*), SERS markedly amplifies the Raman signals of biological markers, thereby enabling applications in cancer diagnosis and therapy, drug monitoring, and infectious disease detection (*19*). In parallel, electrophysiological measurements represent a fundamental modality for probing bioelectrical activity, with electrocardiography serving as the clinical gold standard for cardiovascular assessment and electromyography enabling precise evaluation of muscle activity in human-machine interfaces (*6*, *25*–*27*). SERS and electrophysiological sensing are inherently complementary in terms of spatiotemporal resolution, target scale, and information dimension. Integrating these modalities into a single, flexible platform holds strong appeal, as it could enable multimodal, multiscale biological information analysis that bridges chemical and electrical domains. Nevertheless, achieving this synergy remains challenging because of intrinsic material constraints and the structural incompatibility between SERS-active and electrophysiological interfaces.

Conventional SERS substrates are typically fabricated from rigid inorganic materials, which lack conformability and fail to adhere effectively to soft or dynamically changing surfaces such as human skin. Moreover, the plasmonic “hotspot” structures responsible for signal enhancement are highly susceptible to mechanical deformation, restricting their deployment in wearable scenarios (*28*, *29*). In contrast, conventional electrophysiological electrodes often rely on conductive gels to reduce skin impedance (*14*, *30*, *31*). These gels tend to dry out over time, may cause skin irritation, and are unsuitable for prolonged monitoring. Flexible dry electrodes, on the other hand, require no skin pretreatment or conductive gel application. They offer improved comfort and better mechanical conformity at the skin-electrode interface, making them more suitable for long-term wearable applications (*26*, *32*–*34*). Despite these advances, there remains an urgent need for an integrated, dual-functional platform that can simultaneously enable highly sensitive SERS detection and long-term, stable acquisition of electrophysiological signals. Such a system must simultaneously deliver mechanical flexibility, robust plasmonic activity, reliable electrical conductivity, strong skin conformability, and compatibility with substrates of varied morphology. Meeting these requirements is particularly critical for diverse application scenarios, ranging from continuous sleep monitoring to advanced human-machine interfaces, where a truly “multimodal interface” is needed to seamlessly bridge optical and electrical signals across spatial and mechanical domains.

Here, we present a vertically aligned Au-Ag nanowire (VA Au-Ag NW) array as a morphology-adaptive, dual-functional conformal sensing platform. Using a template-guided self-assembly strategy that requires neither high-temperature processing nor complex transfer steps, highly ordered VA Au-Ag NW arrays can be directly fabricated on diverse one-dimensional (1D)–3D substrates, including 1D nano/micro needles, 2D elastic films, and 3D porous sponges. When integrated on elastic 2D films, the VA Au-Ag NWs serve as FlexoSERS substrates, delivering high sensitivity with excellent spatial uniformity [relative standard deviation (RSD) = 7.20%]. SERS performance remains stable under 100% strain and after 2500 repeated stretching cycles, indicating outstanding mechanical durability and strain-insensitive performance. When grown on 3D porous sponges, the VA Au-Ag NWs function as gel-free dry electrodes capable of long-term, high-quality electrocardiogram (ECG) and electromyogram (EMG) signal acquisition without the need for conductive gels. Continuous ECG monitoring combined with a deep learning model enables accurate identification of sleep and wake states, demonstrating potential for real-time physiological state evaluation. Meanwhile, the high EMG sensitivity allows precise detection of subtle finger motions, keystrokes, and mouse clicks, providing an additional noninvasive signal channel for advanced human-machine interaction.

## RESULTS

### Morphology-adaptive design of VA Au-Ag NW arrays for dual plasmonic-electronic functionality

**Fig. 1.**
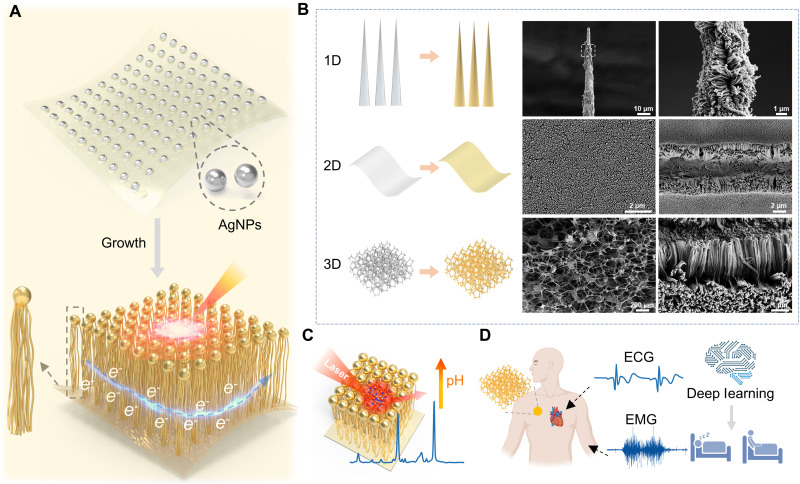
Design, structural versatility, and multifunctional applications of VA Au-Ag NWs arrays. (**A**) Schematic illustration of the seed-mediated growth strategy. (**B**) VA Au-Ag NWs conformally grow on 1D, 2D, and 3D substrates. (**C**) Application of a 2D VA Au-Ag NW array as a pH-responsive FlexoSERS platform. Figures created with Microsoft PowerPoint, Blender, and 3ds Max. (**D**) Application of the 3D VA Au-Ag NW–integrated sponge as a dry, gel-free electrode for electrophysiological monitoring. Created in BioRender. Chen, Y. (2026) https://BioRender.com/a77766z.

Figure 1 illustrates the design concept, structural adaptability, and multimodal applications of VA Au-Ag NW arrays, which integrate plasmonic and electronic functionalities within a structurally adaptive architecture. The fabrication strategy involves using silver nanoparticles (AgNPs) as nucleation centers to direct the anisotropic, vertical growth of bimetallic Au-Ag NWs. This hierarchical configuration naturally integrates dual functionality: The upper region forms dense plasmonic hotspots that drive sensitive SERS performance, while the lower region establishes conductive pathways for efficient electron transport and electrophysiological signal acquisition (Fig. 1A). A defining feature of this system is its morphology-adaptive capability. The VA Au-Ag NWs can be conformally grown on substrates spanning 1D nanoneedles and 2D elastic films to 3D porous sponge (Fig. 1B). In addition to these, uniform nanowires growth was also obtained on practical substrates such as microneedle arrays (fig. S1) and medical cotton swabs (fig. S2), demonstrating excellent geometric versatility. This geometric versatility enables seamless integration with soft, flexible, and topographically complex materials, offering a universal platform for constructing multifunctional biointerfaces.

The dual functionality of VA Au-Ag NWs emerges as its geometry adapts to different application contexts. When integrated with 2D elastic films, the arrays serve as FlexoSERS substrates for chemical detection, leveraging high-density, uniform nanowire coverage to achieve sensitive and reproducible molecular analysis (Fig. 1C). In contrast, when embedded in a 3D porous sponge, the arrays function as dry bioelectrodes, enabling high-quality, gel-free acquisition of electrophysiological signals such as ECG and EMG (Fig. 1D). Benefiting from its porous structure and intimate skin contact, the nanowire-functionalized sponge enabled stable signal acquisition without conductive gels. Coupled with deep learning algorithms, the recorded biosignals can be further classified to assess physiological states such as sleep stages, illustrating the potential of the system for intelligent and continuous health monitoring. Together, these results highlight the morphology-adaptive and dual-functional nature of VA Au-Ag NW arrays, which unite plasmonic and electronic sensing within a single materials platform and provide a scalable pathway toward next-generation multimodal biointerfaces.

### Morphological characterization of the VA Au-Ag NWs

**Fig. 2.**
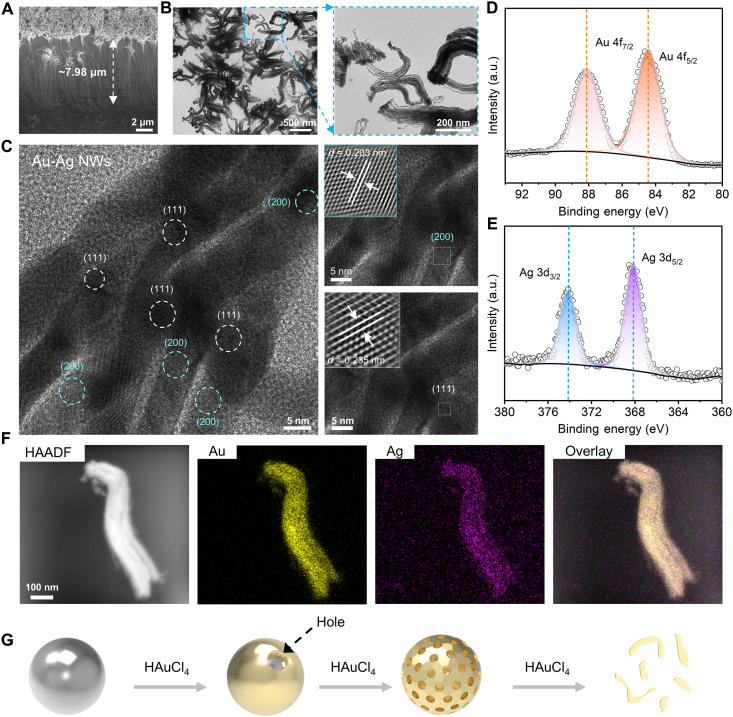
Morphological characterization and formation mechanism of the VA Au-Ag NWs. (**A**) Cross-sectional SEM image. (**B**) TEM images. (**C**) High-resolution TEM image and corresponding lattice analysis. (**D** and **E**) XPS spectra of Au 4f and Ag 3d. (**F**) EDS elemental mapping images including high-angle annular dark-field (HAADF), Au, Ag, and overlay. (**G**) Schematic illustration of the galvanic replacement process. Image created with Microsoft PowerPoint and 3ds Max. a.u., arbitrary units.

The morphology-adaptive nature and dual functionality of the VA Au-Ag NWs originates from their unique hierarchical structure and alloyed composition (Fig. 2). As shown in Fig. 2 (A and B), scanning electron microscopy (SEM) and transmission electron microscopy (TEM) images reveal a hierarchical architecture, characterized by multiple nanowires connected at a common head. Each individual nanowire exhibits an average diameter of 8.33 ± 0.96 nm (fig. S3), with an aspect ratio reaching up to ~957.88. Such ultrahigh aspect ratios and vertical ordering provide large accessible surface areas and highly reproducible optical and electrical interfaces, which are essential for dual-mode sensing.

The crystalline and alloyed nature of the nanowires was confirmed by structural and compositional analyses. The x-ray diffraction (XRD) pattern of the VA Au-Ag NWs in fig. S3 proves high crystallinity and matches well with the XRD patterns of Au and Ag (JCPDS 04-0784 and 04-0783), owing to the similar lattice constants of Au and Ag (*35*). The crystallinity of VA Au-Ag NWs is also demonstrated by the high-resolution TEM image in Fig. 2C, which shows distinct lattice fringes. The observed lattice spacings of 0.203 and 0.235 nm correspond to the (200) and (111) planes of a face-centered cubic lattice of Au or Ag, respectively. X-ray photoelectron spectroscopy (XPS) is used to analyze the chemical state of VA Au-Ag NWs. As shown in Fig. 2 (D and E), the Au 4f and Ag 3d spectra are both decomposed into two characteristic spin-orbit peaks. The Au 4f7/2 and 4f5/2 peaks are located at a binding energy of 84.4 and 88.1 eV, and the Ag 3d5/2 and 3d3/2 peaks occur at 368.1 and 374.1 eV. The gap between the 4f7/2 and 4f5/2 peaks of Au is 3.7 eV, and the gap between the 3d5/2 and 3d3/2 peaks of Ag is 6.0 eV, which are the same as the values of zero-valent Au and Ag (*36*). Combining the uniform and highly overlapping distribution of Au and Ag elements revealed by energy-dispersive x-ray spectroscopy (EDS) mapping (Fig. 2F and fig. S5), the nanowires are supported to exist in the form of a homogeneous Au-Ag alloy. Meanwhile, the line scanning in the SEM image further confirms that the distribution trends of Au and Ag elements were completely consistent (fig. S5). These results collectively verify the formation of uniform Au-Ag alloy NWs, in contrast to core-shell or phase-separated morphologies, ensuring homogeneous and stable physicochemical properties across large-scale arrays.

The formation pathway of the VA Au-Ag NWs can be explained by the following mechanism (Fig. 2G) (*37*). Initially, AgNPs were immobilized on the substrate surface via silanization. The growth of nanowires was then initiated in an aqueous solution containing HAuCl_4_ (Au^3+^ precursor) and ascorbic acid (AA; reducing agent). At the initial stage, galvanic replacement occurred at high-energy sites on the Ag surface, where Ag was oxidized to form nanoholes and AuCl_4_^−^ was reduced to Au. The deposited Au formed a discontinuous shell, which gradually alloyed with Ag to form a core-shell structure. With excess HAuCl_4_, Ag was selectively leached from the shell, enlarging holes and eventually collapsing the structure into Au-rich nanofragments. Notably, this replacement process occurred in a reducing environment provided by AA, which effectively suppressed the formation of AgCl by preventing the interaction between Ag^+^ and Cl^−^. As a result, the residual Ag^+^ and Au^3+^ ions were coreduced on the Au-rich fragments, which served as active nucleation sites. This coreduction, combined with a vertically oriented growth mechanism, ultimately gave rise to the formation of VA Au-Ag NWs. To further validate the proposed formation mechanism of VA Au-Ag NWs, we conducted a series of control experiments by replacing AgNPs with AuNPs and gold nanostars as seeds. In these experiments, AgNPs were added only during the final growth stage. As shown in fig. S6, vertical nanowires growth was still observed under both conditions. Simultaneously, EDS analysis confirmed that the resulting nanowires were composed of both Au and Ag, further supporting the proposed growth mechanism of VA Au-Ag NWs.

### SERS performance of the VA Au-Ag NWs on elastic 2D films

**Fig. 3.**
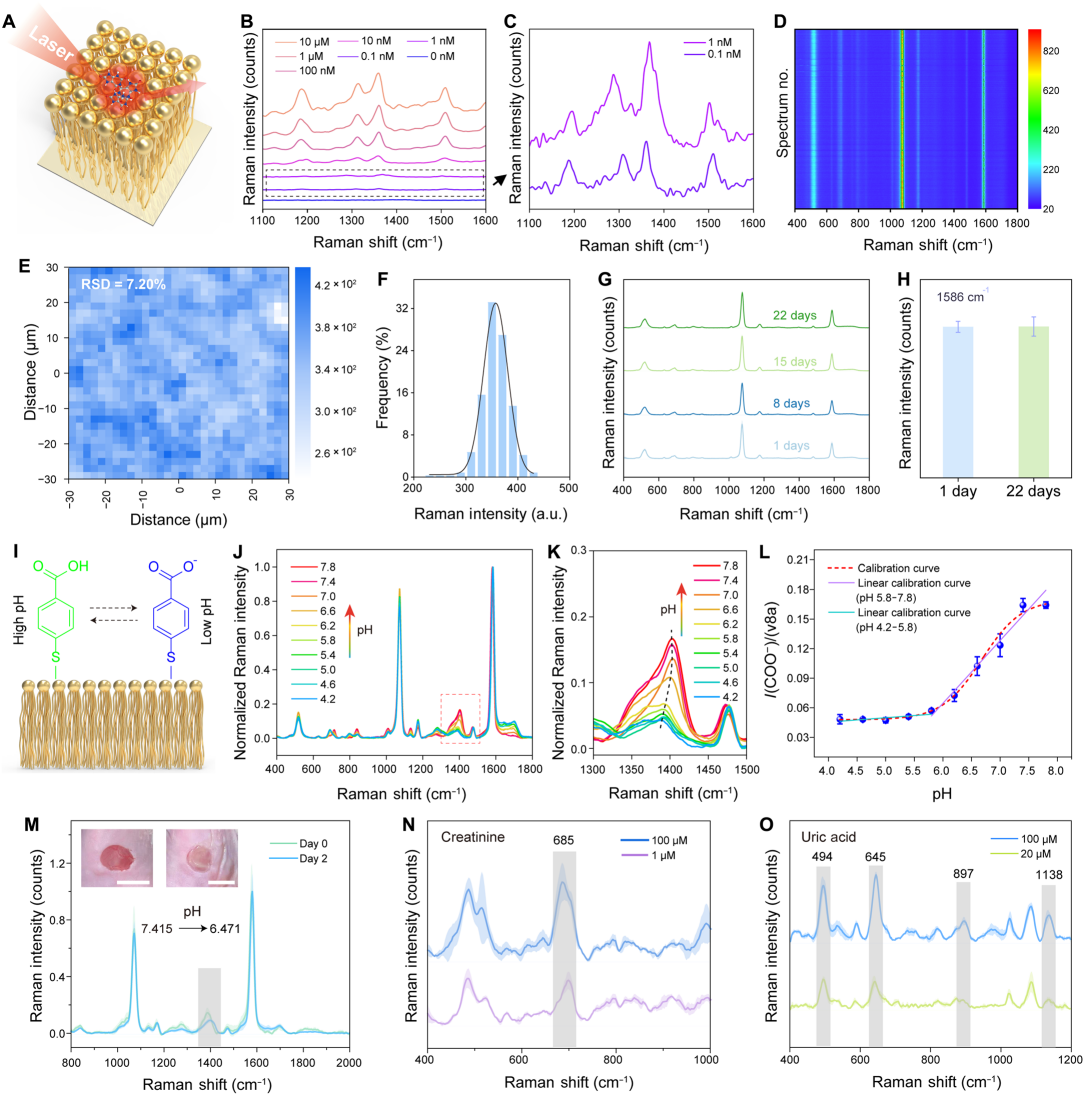
SERS performance of the VA Au-Ag NWs on a 2D elastic film. (**A**) Schematic illustrating the VA Au-Ag NWs as a 2D FlexoSERS platform. (**B**) SERS spectra of R6G on the sensor at various concentration ranges ranging from 0.1 nM to 10 μM. (**C**) SERS spectra of R6G at concentrations of 0.1 and 1 nM. (**D**) SERS contour of 900 spectra of 4-MBA acquired from 900 different locations on a single substrate. (**E**) Raman intensity mapping (~1078 cm^−1^) over a 60 μm–by–60 μm area (3600 μm^2^). (**F**) Histogram of Raman intensity at ~1078 cm^−1^ extracted from the (E). (**G** and **H**) Evaluation of SERS stability. (**I**) Schematic illustration of pH sensing. Image created with Microsoft PowerPoint and 3Ds Max. (**J**) SERS spectra of 2D VA Au-Ag NW FlexoSERS film in McIlvaine buffer with pH from 4.2 to 7.8. (**K**) A closer look of the Raman intensity of the νCOO^−^ mode at ~1417 cm^−1^ when the pH changes. (**L**) Calibration curve for the relative intensity of the νCOO^−^ to the benzene ring ν8a mode against pH. (**M**) In vitro pH monitoring of bacterial infections in a murine wound model. (**N** and **O**) Label-free SERS detection of creatinine and uric acid. Image created with Microsoft PowerPoint and 3ds Max.

When VA Au-Ag NWs are grown on 2D elastic films, they form a morphology-adaptive plasmonic network that serves as a robust FlexoSERS platform (Fig. 3A). Following removal of the surface-bound 4-mercaptobenzoic acid (4-MBA) ligands by using sodium borohydride solution and plasma treatment, the SERS sensitivity was evaluated using rhodamine 6G (R6G) as a Raman reporter. As shown in Fig. 3 (B and C) and fig. S7, the 2D VA Au-Ag NW FlexoSERS film enabled sensitive detection of R6G molecules over a wide concentration range from 0.1 nM to 10 μM. The intrinsically hydrophobic surface (fig. S8) further promoted analyte preconcentration, amplifying sensitivity without requiring additional surface engineering (*38*). Moreover, by tuning the seed concentration (1000 × 10^−7^ to 15.625 × 10^−7^ M), VA Au-Ag NW FlexoSERS film with varying surface densities were obtained (fig. S9), which decreased with lower seed concentrations. Correspondingly, the SERS performance showed a nonmonotonic trend: poor at high concentrations, sharply enhanced at intermediate levels, and dropped again at very low concentrations (fig. S10). To further understand this behavior, a finite element method (FEM) based on a nanosphere array model was conducted. The results revealed that electromagnetic (EM) hotspots first intensified and then weakened with increasing interparticle spacing (fig. S11), explaining the observed SERS variation.

The conformal nanowire architecture also ensured high reproducibility. Given the abundant 4-MBA molecules inherently adsorbed on the NW surface, SERS uniformity of the 2D VA Au-Ag NW FlexoSERS film was assessed directly. Figure 3D presents the contour of 900 SERS spectra of 4-MBA acquired from 900 different locations on a single substrate, revealing exceptional spectral reproducibility across macroscale areas. In addition, Raman intensity mapping over a 60 μm–by–60 μm area (3600 μm^2^) further confirmed spatial uniformity, with the intensity at ~1078 cm^−1^ yielded a Gaussian intensity distribution with a low RSD (=7.20%) (Fig. 3E). To assess stability, the 2D VA Au-Ag NW FlexoSERS film was stored in ambient air for 22 days. Negligible variation in SERS intensity was observed (Fig. 3, G and H, and fig. S12), indicating excellent retention of SERS activity over time.

Beyond model reporters, the 2D VA Au-Ag NW FlexoSERS film exhibited multifunctional biochemical sensing capabilities. The surface-enriched 4-MBA molecules, a well-established pH-sensitive reporter (*16*, *39*, *40*), endow the 2D VA Au-Ag NW FlexoSERS film with intrinsic pH responsiveness. Figure 3I and fig. S13 illustrate the pH sensing mechanism: The carboxyl group of 4-MBA exhibits pH-dependent ionization states, which modulate the symmetric COO^−^ stretching mode (νCOO^−^). Stacked SERS spectra collected from pH 4.2 to 7.8 (Fig. 3J) reveal that the νCOO^−^ peak (~1417 cm^−1^) intensifies with increasing pH, especially above pH 5.8, due to increased deprotonation of carboxyl groups (Fig. 3K). Calibration curves of intensity ratios *I*(νCOO^−^)/*I*(ν8a) and *I*(νCOO^−^)/*I*(ν1) as a function of pH exhibit a sigmoidal trend (Fig. 3L and fig. S14), with enhanced sensitivity in the pH range of 5.8 to 7.8 and weaker correlation at lower pH (pH 4.2 to 5.8), consistent with previous reports. To demonstrate its practical applicability, we used the 2D VA Au-Ag NW FlexoSERS film for in vitro pH monitoring of bacterial infections in a murine wound model. While physiological tissue typically maintains a neutral pH, bacterial proliferation leads to localized acidification (*41*). The SERS results showed that at the early stage of infection, the wound pH was approximately 7.415, which decreased to 6.471 after 2 days of infection, demonstrating the potential of 2D VA Au-Ag NW FlexoSERS film for real-time monitoring of bacterial infection progression (Fig. 3M).

Furthermore, the 2D VA Au-Ag NW FlexoSERS film enabled label-free detection of clinically relevant biomolecules in human sweat and urine, such as creatinine and uric acid, demonstrating its multifunctional biosensing capabilities. Figure 3N presents the SERS spectra of creatinine solutions at 100 and 1 μM. The characteristic peak at 685 cm^−1^ is assigned to the intramolecular vibrational mode of the lactam ring (*42*). Figure 3O displays the SERS spectra of uric acid solutions at 100 and 20 μM, where distinct peaks at 645, 897, and 1138 cm^−1^ correspond to skeletal ring deformation, N─H bending, and C─N stretching vibrations of uric acid (*16*, *20*, *43*, *44*), respectively. The peak observed at 495 cm^−1^ arises from the in-plane ring deformation and C─N bending of uric acid, along with a contribution from the Si─O─Si stretching vibration of the poly(dimethylsiloxane) (PDMS) substrate (*45*).

### Stretchability and mechanical durability of 2D VA Au-Ag NW FlexoSERS films

**Fig. 4.**
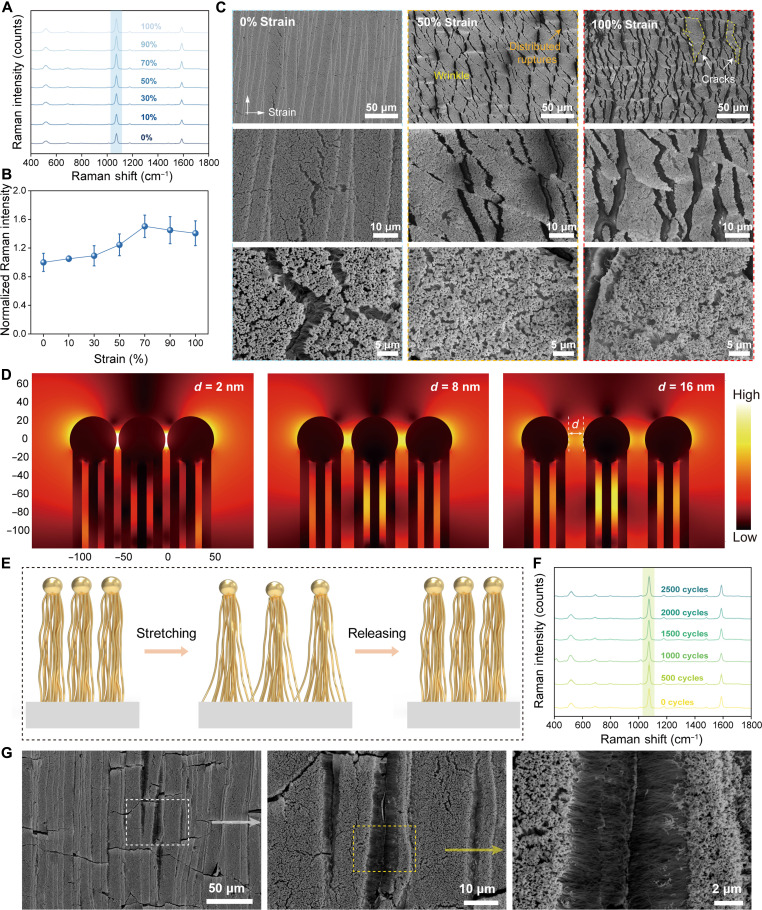
Stretchability of the 2D VA Au-Ag NW FlexoSERS films. (**A**) Raman spectra of the 2D VA Au-Ag NW FlexoSERS film under tensile strains ranging from 0 to 100%. (**B**) Normalized Raman intensity of the 2D VA Au-Ag NW FlexoSERS film under different strains that extracted from (A). (**C**) SEM morphology of the 2D VA Au-Ag NW FlexoSERS film under various tensile states. (**D**) Local EM field distribution around the 2D VA Au-Ag NWs with varying internanowire distances. . (**E**) Schematic illustrating the deformation recovery process during repeated stretch-release cycles. (**F**) SERS spectra of the 2D VA Au-Ag NW FlexoSERS film during 2500 cycling test under 50% stretch. (**G**) SEM images of the 2D VA Au-Ag NW FlexoSERS film after 3000 cycles. Image created with Microsoft PowerPoint and 3ds Max.

To evaluate their adaptability under mechanical deformation, we systematically investigated the stretchability of the 2D VA Au-Ag NW FlexoSERS films. Raman spectra of 4-MBA collected under tensile strains from 0 to 100% showed negligible spectral deviations in vibrational fingerprints, with the characteristic band at ~1078 cm^−1^ maintaining strong intensity across the entire strain range (Fig. 4A). Quantitative analysis revealed that the Raman intensity initially increases with applied strain before exhibiting a slight decrease at higher strain levels (Fig. 4B), yet overall stability was preserved across the full strain range.

To elucidate the underlying stretchability mechanism, we examined the SEM morphology of the 2D VA Au-Ag NW FlexoSERS film under various tensile states (Fig. 4C). With increasing strain, the film exhibited distributed ruptures (*46*, *47*) and cracks aligned parallel to the strain direction, with the density of these features increasing notably as strain was amplified. Conversely, in the direction perpendicular to the applied strain, the film surface developed wrinkles due to compressive strain resulting from the Poisson’s effect (*48*). High-resolution imaging further confirmed that under such compressive strain, the film surface exhibited a more densified configuration, leading to reduced internanowire spacing. FEM simulations of EM field distributions confirmed this mechanism (Fig. 4D). The results show that reduced internanowire distance leads to intensified EM “hotspots” within the nanogaps, enhancing the local EM field. This enhancement mechanism, in conjunction with the morphological observations from the SEM images under tensile conditions, collectively explains the Raman intensity variation trends observed in Fig. 4B.

To assess mechanical durability, we conducted cyclic stretching tests on the 2D VA Au-Ag NW FlexoSERS film. Figure 4E schematically illustrates the deformation-recovery process during repeated stretch-release cycles. The VA Au-Ag NWs are anchored at their base into the elastic substrate (*47*), allowing their spatial configuration to reversibly respond to substrate elongation and relaxation. Figure 4F demonstrates that the SERS spectral features of 4-MBA remained well defined after 2500 stretch-release cycles, indicating preserved sensing capability under prolonged tensile loading. The Raman intensity at ~1078 cm^−1^ showed a negligible decrease with increasing cycle number (fig. S15). Postcycling SEM examination (Fig. 4G) revealed minimal irreversible cracking after 2500 cycles, which accounts for the observed negligible decrease in Raman intensity. Notably, the overall microscopic morphology exhibited no substantial alteration compared to its unstretched state, confirming the superior tensile durability and maintained optical performance of the FlexoSERS platform. These results highlight the morphology-adaptive nature of the 2D VA Au-Ag NW films, which combine plasmonic responsiveness with mechanical resilience, ensuring consistent SERS performance even under extreme and repeated mechanical deformation.

### Biocompatibility evaluation of 2D VA Au-Ag NWs

To evaluate the biocompatibility of the VA Au-Ag NWs, both acridine orange/ethidium bromide (AO/EB) staining and Cell Counting Kit-8 (CCK-8) assays were performed using 3T3 cells. As shown in fig. S16, the AO/EB-stained fluorescence images revealed a large number of viable cells in both the blank and VA Au-Ag NW groups, indicating negligible cytotoxicity. In addition, the CCK-8 assay results (fig. S17) showed a continuous increase in the optical density (OD) values of 3T3 cells from 24 to 72 hours. The cell viability of the VA Au-Ag NW group remained ~100% compared with the blank control and even exhibited a slight enhancement at 48 hours. These findings demonstrate that the VA Au-Ag NWs have good biocompatibility and can support normal cell proliferation during prolonged culture.

### Electrophysiological monitoring enabled by VA Au-Ag NW elastronics on a 3D sponge

**Fig. 5.**
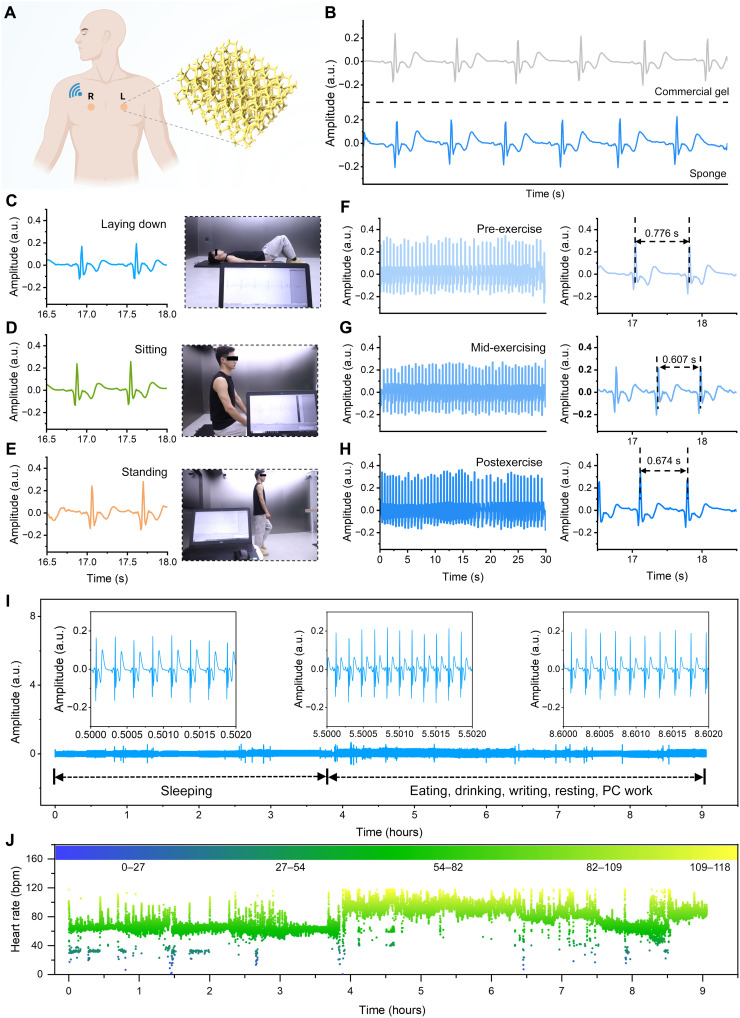
ECG monitoring enabled by VA Au-Ag NWs on a 3D porous sponge. (**A**) Schematic diagram of the wireless ECG recording setup. Created in BioRender. Chen, Y. (2026) https://BioRender.com/inbqo7j. (**B**) Comparison of ECG signals recorded using the 3D sponge/NW elastronic electrodes (blue) versus a commercial gel electrode (gray). (**C** to **E**) ECG signal recordings in different body positions: (C) laying down, (D) sitting, and (E) standing. Insets show corresponding postures during measurement. (**F** to **H**) ECG signal recordings during pre- (F), mid- (G), and postexercise (H) stages. Right shows magnified ECG segments highlighting heart rate changes, with measured R-R intervals of 0.776, 0.607, and 0.674 s, respectively. (**I**) Continuous ECG monitoring over 9 hours using 3D sponge/NW elastronic electrodes during daily activities, including sleeping, eating, drinking, writing, resting, and PC work. Insets show magnified signal segments during different time intervals, indicating high-fidelity signal retention. (**J**) Heart rate variation calculated from the ECG signals over the 9-hour monitoring period. bpm, beats per minute.

The morphology-adaptive elastronic architecture of VA Au-Ag NWs was further exploited by integrating them onto a 3D porous sponge to construct a dry electrode (3D sponge/NWs) for electrophysiological monitoring. Owing to the intrinsic softness and elasticity of the sponge, the resulting elastronic dry electrode exhibits excellent mechanical compliance, capable of withstanding at least 360° twisting without destruction (fig. S18). The skin-electrode impedance of the 3D sponge/NW elastronic electrode was measured to be 183.97 kΩ at 100 Hz, slightly higher than that of a commercial gel electrode (65.16 kΩ) (fig. S19). Nevertheless, both values were sufficiently low to ensure reliable electrophysiological signal acquisition, as supported by prior studies (*30*, *49*). Figure 5A illustrates the electrode placement for ECG recording, where two 3D sponge/NW elastronic electrodes were attached to the chest and connected to a portable wireless module equipped with Bluetooth for real-time data transmission. As shown in Fig. 5B, the elastronic electrode successfully captured characteristic ECG waveforms comparable to those obtained with commercial hydrogel electrodes.

To evaluate the adaptability of the elastronic electrodes under diverse physiological conditions, ECG signals were recorded from the subject under seven conditions, including lying, sitting, standing, walking, and across pre-, mid-, and postexercise stages, as shown in Fig. 5 (C to H) and fig. S20. Continuous and stable R-wave signals were recorded in all cases, and noticeable changes in the R-R intervals were observed across the pre-, mid-, and postexercise stages, indicating the high responsiveness and robustness in capturing physiological state transitions. To assess the impact of sweat, an experiment was conducted to examine the electrode performance during sweat-induced conditions caused by intense exercise. Even after 10 min of vigorous activity, the electrode maintained clear and distinguishable ECG waveforms (fig. S21), demonstrating the robustness of signal quality against sweat-induced interference.

Long-term performance was also systematically evaluated. As shown in fig. S22, ECG signals collected on days 1, 4, and 10 remained stable, with enlarged waveform segments revealing consistent morphology over time, confirming its signal retention capability and long-term reliability. In addition, the signal-to-noise ratio (SNR) remained high (~16 dB) after 10 days of use, with no notable deterioration compared to earlier days (fig. S22). Furthermore, continuous monitoring over a 9-hour period during daily activities such as sleeping, eating, writing, and resting demonstrated the electrodes’ capacity for high-fidelity, uninterrupted recording (Fig. 5I). Figure 5J summarizes heart rate variations over the 9-hour duration, clearly reflecting dynamic fluctuations under different activity states and showcasing the practical potential of this electrode for wearable health monitoring. Moreover, by integrating bidirectional long short-term memory (BiLSTM) network, the 9-hour ECG dataset further enabled accurate classification of sleep and wake states (figs. S23 and S24), underscoring the intelligent sensing potential of the elastronic system.

**Fig. 6.**
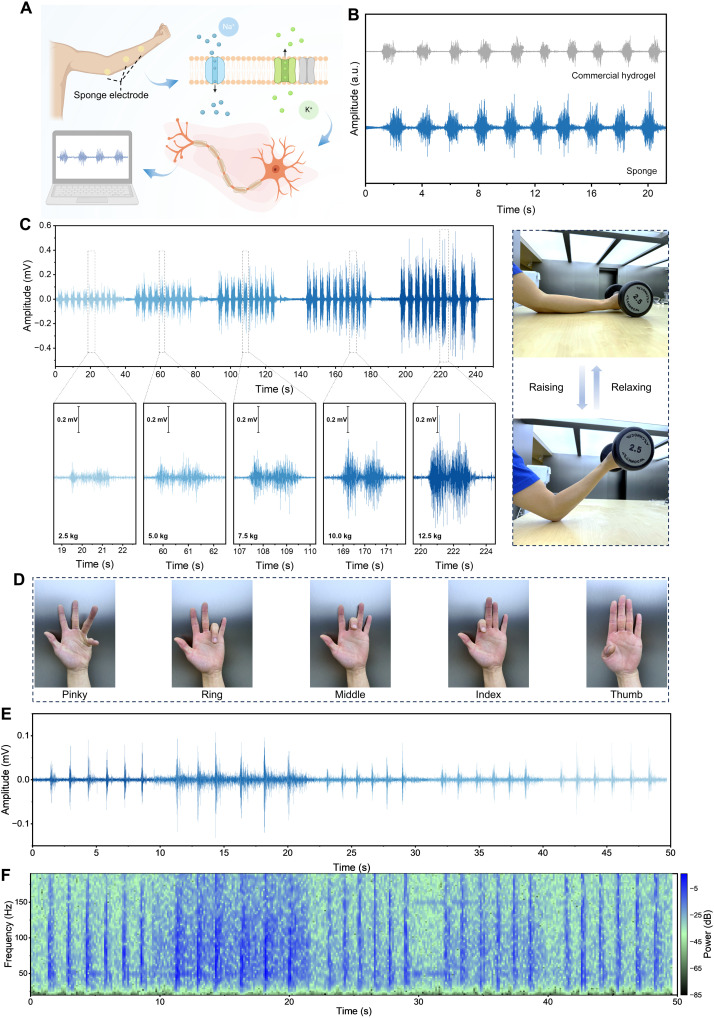
EMG monitoring enabled by VA Au-Ag NWs on a 3D sponge. (**A**) Schematic diagram of the EMG acquisition mechanism by 3D sponge/NW elastronic electrode. Created in BioRender. Chen, Y. (2026) https://BioRender.com/inbqo7j. (**B**) Comparison of EMG signals during fist clenching, recorded using the 3D sponge/NW dry electrode (blue) and a commercial gel electrode (gray). (**C**) EMG signals recorded during repetitive forearm lifting and relaxing tasks with gradually increasing weights of dumbbells from 2.5 to 12.5 kg. Insets show magnified EMG segments corresponding to different weight loads (2.5, 5.0, 7.5, 10.0, and 12.5 kg). Right: photographic illustration of the arm movement cycle during weight lifting and relaxation. (**D**) Photos of the flexion/extension of different fingers. (**E**) EMG signals recorded during the individual finger movements shown in (D). (**F**) Time frequency spectrogram of the EMG signals in (E).

The versatility of the elastronic electrode was further validated in EMG monitoring through a series of physiological motion tests, including forearm muscle contractions under varying loads and fine finger movements. As illustrated in Fig. 6A, the dry electrode records muscle activity by capturing local field potentials generated by the superposition of action potentials from the excited neurons. The porous structure of the 3D sponge/NW elastronic electrode enhances the effective contact with the skin surface, thereby reducing interfacial impedance and enabling continuous acquisition of EMG signals. Comparative experiments were carried out between the 3D sponge/NW elastronic electrode and commercial gel electrodes (Fig. 6B). Under identical motion conditions, the elastronic electrode yielded EMG signals with higher amplitudes and more pronounced periodicity. The SNR was ~11 dB, comparable to that of commercial gel (fig. S25). To further evaluate signal quality, we conducted both time-domain and frequency-domain analyses, the standard methodologies for characterizing muscle activity. Fourier transform of the EMG signals revealed that both electrodes captured dominant spectral components within the 20- to 150-Hz range, with similar mean power frequency and median frequency values (fig. S25), suggesting comparable neuromuscular fidelity.

We further assessed the responsiveness of elastronic electrode to varying forearm muscle contraction intensities. Figure 6C displays the EMG signals recorded as the subject lifted dumbbells of increasing weight (2.5 to 12.5 kg). The signal amplitude increased proportionally with the load, reflecting enhanced muscle activation. Enlarged insets of signal segments clearly show the repeatable discharge patterns associated with each lifting cycle, underscoring the reliability of the electrode for dynamic intensity tracking.

In addition, the elastronic electrode was used to detect EMG signals during finger flexion and extension (Fig. 6D). To assess spatial resolution, individual finger-lifting tasks were performed sequentially, with corresponding EMG responses recorded (Fig. 6E). Distinct and patterned signal fluctuations were observed for each finger, demonstrating the capability to resolve localized muscle activity and its suitability for fine-motor recognition and hand function assessment. Time-frequency analysis of these signals (Fig. 6F) revealed transient high-power bursts aligned with each finger movement, primarily distributed within 0 to 250 Hz, consistent with characteristic EMG spectra. This confirms the elastronic electrode’s high-fidelity capture of dynamic muscle activity.

To evaluate long-term stability and motion responsiveness, continuous EMG monitoring was conducted over a ~10-min session involving repetitive finger bending and straightening. The subject, equipped with the 3D sponge/NW elastronic electrode, performed rhythmic finger flexion-extension motions. As shown in fig. S26, the recorded EMG signals maintained stable waveforms and consistent amplitudes throughout the 600-s monitoring period, highlighting the electrode’s excellent durability and anti-interference capability. Zoomed-in views of three representative motion cycles (~28 to 34, 308 to 314, and 540 to 546 s) revealed highly reproducible EMG patterns for each finger movement, validating the elastronic electrode’s reliability and consistency for motion recognition and functional assessment.

High-sensitivity detection was also demonstrated through fine motor tasks, such as keyboard typing and mouse clicking. Although these subtle actions, typically involving minimal muscle contraction, they still generated clearly distinguishable EMG signals (fig. S27). Specifically, the electrodes successfully captured sharp, high-amplitude spikes corresponding precisely to each key press or mouse click. Furthermore, enlarged signal segments (fig. S27), along with synchronized motion images, confirmed accurate temporal alignment and signal clarity. Collectively, these results demonstrate that the VA Au-Ag NW–based morphology-adaptive elastronics enable dual-function electrophysiological monitoring with high fidelity, robustness, and long-term stability, underscores their promise for next-generation wearable health monitoring, neurorehabilitation, and human-machine interaction.

## DISCUSSION

This work demonstrates a morphology-adaptive, dual-functional elastronic sensing platform based on VA Au-Ag NW arrays, which unifies plasmonic FlexoSERS and electrophysiological monitoring within a single elastronic material. The VA Au-Ag NW arrays can be conformally integrated onto diverse 1D to 3D substrates, including microneedles, elastic films, and porous sponges, without requiring complex fabrication processes. On 2D elastic substrates, the VA Au-Ag NW arrays deliver high-sensitivity, uniform, and mechanically durable FlexoSERS performance, while on 3D porous sponges, they serve as robust elastronic electrodes for stable, long-term ECG and EMG signal acquisition. The recorded multimodal signals empower downstream applications such as pH sensing, sleep-wake state classification, and fine motion recognition, underscoring the multifunctionality and translational potential of the system.

Together, these results establish a morphology-adaptive elastronic strategy that bridges chemical and electrical biosensing. The demonstrated versatility across 1D-3D interfaces further points to broad opportunities for integrated biochemical-electrophysiological sensing in more complex physiological environments. Overall, this work offers a unified materials framework for advancing next-generation wearable health monitoring, personalized diagnostics, and human-machine interaction.

## MATERIALS AND METHODS

### Chemicals and reagents

Hydrogen tetrachloroaurate(III) trihydrate (HAuCl_4_·3H_2_O, ≥49.0% Au basis), phosphate-buffered saline tablet, L(+)-ascorbic acid (≥99%), sodium citrate tribasic dihydrate (≥99%), uric acid (≥99%), 3-aminopropyltriethoxysilane (APTES, ≥98%), 4-MBA (≥90%), creatinine (C_4_H_7_N_3_O, ≥98%), and sodium borohydride (NaBH_4_, ≥99%) were purchased from Sigma-Aldrich. Citric acid (C_6_H_8_O_7_, ≥99.5%) was purchased from Macklin. Silver nitrate (AgNO_3_, ≥99%) was purchased from Aladdin. Sodium phosphate dibasic dodecahydrate (Na_2_HPO_4_·12H_2_O, analytical grade) and ethanol (analytical grade) were purchased from Greagent. SYGARD 184 was purchased from DOWSIL. A kitchen nanosponge (with a density of 8 × 10^−3^ g cm^−3^) was purchased from Hangzhou Mu Zhi Yun Craft Co. Ltd., China.

### Synthesis of citrate-stabilized AgNPs

The citrate-stabilized AgNPs were synthesized according to previously reported methods with some modifications (*50*). First, 2- to 4-nm silver nanoseeds were prepared. Seventy-five milliliters of deionized water was mixed with 20 ml of a 1% sodium citrate solution and heated to 80° to 82°C. Under vigorous stirring, 1.7 ml of a 1% AgNO_3_ solution was added, followed by a rapid one-time addition of 2 ml of a freshly prepared 0.1% NaBH_4_ solution. After stirring for 1 hour, the mixture was naturally cooled to room temperature and adjusted to a final volume of 100 ml.

Next, 20- to 25-nm silver nanospheres were prepared on the basis of the silver nanoseeds. Seventy-five milliliters of deionized water was mixed with 2 ml of a 1% sodium citrate solution and heated to boiling. Under vigorous stirring, 10 ml of the 4-nm silver nanosphere solution and 1.7 ml of a 1% AgNO_3_ solution were sequentially added. The solution was then stirred for 1 hour under reflux conditions. Afterward, 2 ml of a 1% sodium citrate solution and 1.7 ml of AgNO_3_ solution were added and stirred under reflux for an additional hour. This step was repeated twice. Last, the mixture was naturally cooled to room temperature and adjusted to a final volume of 100 ml.

### Preparation of 2D elastic substrate

A mixture of SYLGARD 184 silicone elastomer base and its curing agent was mixed in a 10:1 mass ratio, thoroughly stirred, and degassed under vacuum to eliminate air bubbles. The mixture was then spin-coated onto the surface of a petri dish at a predetermined speed (500 rpm) for 30 s to form a uniform elastic substrate. Last, the prepared substrate was placed in an oven, cured for 2 hours, and then removed for later use.

### Modification of 3D sponge substrate

The commercial nanosponges were cut into thin sheets of specific dimensions (2 cm by 1.5 cm by 0.2 cm) and ultrasonically cleaned in ethanol for 5 min. The cleaned sponge sheets were then immersed in a mixture of PDMS precursor and curing agent (in a 20:1 mass ratio) and treated under vacuum to ensure thorough impregnation. Excess PDMS precursor and curing agent within the sponge were then removed by extrusion, followed by a secondary removal using a centrifuge at 2000 to 2500 rpm for 3 to 5 min to further eliminate any remaining PDMS. Last, the modified sponge was placed in an oven, cured for 1 hour, and then removed for later use.

### Modification of substrate surface with AgNPs

The 1D to 3D substrates were subjected to hydrophilic treatment using a plasma cleaner for a defined period, depending on the material type. Specifically, glass substrates were treated for 3 min, PDMS substrates for 5 min, and sponge materials for 15 min. Subsequently, the hydrophilic-treated substrates were immersed in a 4 mM APTES solution for 2 hours to be functionalized with amino groups. After functionalization, the substrates were rinsed thoroughly and immersed in the prepared AgNPs solution for 2 hours to complete the deposition of the seed layer.

### Fabrication of VA Au-Ag NWs on 1D to 3D substrates

VA Au-Ag NWs were fabricated according to our previous method with some modifications (*15*, *16*, *47*). The same fabrication approach was applied to 1D to 3D substrates. In simple terms, the substrates modified with AgNPs were immersed in a growth solution containing 11.9 mM HAuCl_4_, 1.2 mM 4-MBA, and 29.1 mM L-AA for 30 min. This resulted in the formation of Au-Ag NW forests.

### Characterization

Electron imaging was carried out by Nova Nano SEM450, Zeiss Ultra Plus, and Talos F200X. Optical microscope imaging was carried out by Horiba France Sas Xplora. O_2_ plasma (SUNJUNE PLASMA VP-RS) was used to make the substrate hydrophilic. XPS spectra were obtained by using a Thermo Fisher Scientific 250Xi. XRD measurements were performed by using an Ultima IV. The resistance was measured using the two-point probe method by the digital multimeter (DMM 6500, Keithley). The skin-electrode impedance was tested by a low-frequency ac impedance analyzer (Agilent 4294A, 40 Hz to 110 MHz). All Raman and SERS spectra were recorded by HORIBA XploRA PLUS. For the sensitivity, stability, and stretchability tests, a 785-nm Raman laser was used with an integration time of 10 s and a laser power of 1 mW. For Raman mapping, the same 785-nm laser was used with an integration time of 1 s and a laser power of 1 mW. For pH-dependent SERS measurements, a 638-nm laser was used with an integration time of 10 s and a laser power of 1 mW. For uric acid detection, a 785-nm laser was applied with an integration time of 25 s and a laser power of 1 mW. Similarly, creatinine detection was conducted using a 785-nm laser with an integration time of 25 s and a laser power of 1 mW. The durability assessment involved the application of stretch cycles using the MARK-10, ESM303/ESM303H equipment. All animal studies were performed in accordance with guidelines approved by the Animal Care Committee of Southeast University (20240903001) and in accordance with the Regulations for the Administration of Affairs Concerning Experimental Animals of China.

### Electrophysiological recording

For electrophysiological signal monitoring, 3D sponges with VA Au-Ag NWs were cut into square shapes (1 cm by 1 cm) to serve as an elastronic electrode. To record ECG signals, two square sponges (1 cm by 1 cm) were integrated with metal ECG buttons and placed on the left and right chest of the subject, connected to a commercial wireless module (Bitalino, Portugal). The detailed sensor placement is shown in the Fig. 5A, with “R” and “L” representing the positions of the right and left electrodes, respectively. To record EMG signals, two sponges with Ag/AgCl electrodes were adhered to the brachioradialis muscle as measurement electrodes, while another sponge with an Ag/AgCl electrode was attached to the bone (elbow) as a reference electrode, as shown in Fig. 6A. EMG signals were generated when the subject opened and clenched their fist, bent/extended their arm, and flexed their fingers. All experiments involving physiological signal measurements were reviewed and approved by the Ethics Committee of Southeast University (2023-178-05), and informed consent was obtained from all participants before the experiments.

### ECG signal classification by deep learning

The ECG signal was preprocessed by normalizing it to zero mean and unit variance. The signal was then segmented into 60-s windows, with the first segment labeled as “sleep” (0) and the remaining as “work” (1). Gaussian noise with an SD of 0.01 was added to augment the dataset, which was then randomly shuffled and split into 70% training and 30% testing subsets. The data were converted into sequence format for input into a BiLSTM network. The BiLSTM model, with 150 hidden units and a 20% dropout rate, was trained using the Adam optimizer for 30 epochs with an initial learning rate of 1 × 10^−3^ and a mini-batch size of 64. The model’s performance was evaluated using accuracy, precision, recall, and F1 score, with results displayed alongside a confusion matrix. The trained model was saved for future use.

### FEM simulations

FEM simulations were performed using the COMSOL Multiphysics software. The excitation source was a linearly polarized plane wave incident perpendicular to the *xy* plane. The complex refractive indices (*n* + *ik*) of gold and silver were adopted from (*51*, *52*). Scattering boundary conditions were applied to define the simulation boundaries. For fig. S11, the model consisted of nanospheres with a diameter of 50 nm under periodic boundary conditions. The center-to-center distance between spheres was parametrically swept from −20 to 50 nm. For Fig. 4D, the model was simplified to a configuration composed of one nanosphere and three vertically aligned nanowires. The nanosphere had a diameter of 25 nm, while the nanowires had diameters of 10 nm. The center-to-center distance between the nanosphere and nanowires was parametrically swept from 0 to 20 nm.

### Cell culture and cell viability

Cell culture: The 3T3 cells were cultured in a medium composed of high-glucose Dulbecco’s modified Eagle’s medium supplemented with 10% fetal bovine serum and 1% streptomycin/penicillin. The cells were incubated at 37°C in a humidified atmosphere of 5% CO_2_. Cell viability: The cytocompatibility assessment was conducted using the CCK-8 and AO/EB staining assay. 2D VA Au-Ag NW samples were washed with 75% ethanol three times, exposed to ultraviolet radiation overnight, and then positioned in 96-well plates. Subsequently, a 100-μl suspension of 3T3 cells (2 × 10^4^ cells ml^−1^) was cocultured with the 2D VA Au-Ag NWs. The control group was conducted without 2D VA Au-Ag NWs. Cell proliferation was measured after 24, 48, and 72 hours of culture using CCK-8 assay (Sangon Biotech, Shanghai). At each time point, the culture medium was replaced with 100 μl of fresh medium containing 10 μl of CCK-8 solution, followed by a 1.5-hour incubation at 37°C. To determine the OD value, the absorbance at 450 nm (*n* = 3) was measured by a microplate reader. 3T3 cells (1 × 10^5^) were seeded into 24-well plates and incubated with or without 2D VA Au-Ag NWs for 72 hours. All cells were stained with acridine orange and ethidium bromide (AO/EB, Solarbio) following the manufacturer’s instructions. Live cells will appear uniformly green, and necrotic cells will stain orange. Cells were imaged under a fluorescence microscope (IX51, Olympus) to observe the numbers of live and dead cells.

## Supplementary Materials

### This PDF file includes:

Sections S1 to S4 Figs. S1 to S27
