# Supplementary material for: Morphology-adaptive Au-Ag nanowire elastronics for integrated FlexoSERS and bioelectrical sensing
Source: Sci Adv. 2026 Feb 18;12(8):eaec2162. doi: 10.1126/sciadv.aec2162 (PMC12915605; doi:10.1126/sciadv.aec2162)
Supplement: Supplementary file 1 — Sections S1 to S4 Figs. S1 to S27 [file sciadv.aec2162_sm.pdf]

Science Advances
AAAS

Supplementary Materials for

Morphology-adaptive Au-Ag nanowire elastronics for integrated FlexoSERS and bioelectrical sensing

Heng Zhang *et al.*

Corresponding author: Yi Chen, yichen@seu.edu.cn; Wenlong Cheng, wenlong.cheng@sydney.edu.au; Ning Gu, guning@nju.edu.cn

Sci. Adv. **12**, eaec2162 (2026)
DOI: 10.1126/sciadv.aec2162

This PDF file includes:

Sections S1 to S4
Figs. S1 to S27

Section 1. Fabrication and Morphology Characterization of VA Au–Ag NWs

1.1 VA Au–Ag NWs on Microneedle Array

VA Au–Ag NWs were successfully constructed on the surface of a microneedle array (**fig. S1A**). The PDMS microneedle array mold (10 × 10) was purchased from Taizhou Weixin Medical Technology Co., Ltd., with each microneedle featuring a tip height of 500 μm, a base edge length of 260 μm, and an inter-needle spacing of 600 μm. A mixture of SYLGARD 184 silicone elastomer base and curing agent (10:1 by weight) was poured into the mold, followed by degassing under vacuum and curing in an oven at 80 °C for 2 hours. Subsequently, the growth of VA Au–Ag NWs was carried out according to the established protocol. As shown in **fig. S1B**, VA Au–Ag NWs were uniformly formed on the microneedle array surface.

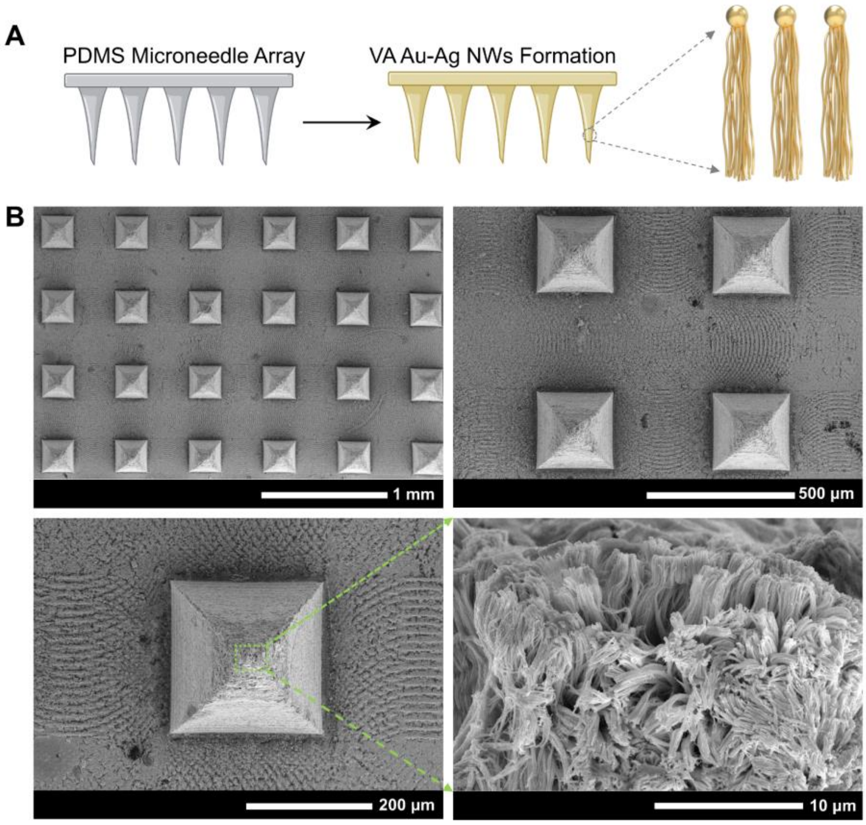

Fig. S1. VA Au–Ag NWs on microneedle array. (A) Schematic illustration of the growth of VA Au–Ag NWs on the surface of the PDMS microneedle array. (B) SEM image showing the successful formation of VA Au–Ag NWs on the microneedle surface. Image created with Microsoft PowerPoint, 3ds Max, and BioRender. Chen, Y. (2026) https://BioRender.com/inbqo7j.

1.2 VA Au–Ag NWs on Medical Cotton Swabs

The growth process of VA Au–Ag NWs on the surface of medical cotton swabs was essentially the same as that on the PDMS microneedle array (**fig. S2A**). The key difference was that, prior to NW growth, the medical cotton swabs were first immersed in a 10:1 (w/w) mixture of PDMS prepolymer and curing agent, followed by vacuum degassing and thermal curing in an oven. As shown in **fig. S2B**, after the growth of VA Au–Ag NWs, the surface of the medical cotton swabs exhibited a characteristic brownish-gold color. SEM imaging further confirmed the successful formation of VA Au–Ag NWs on the swab surface (**fig. S2C**).

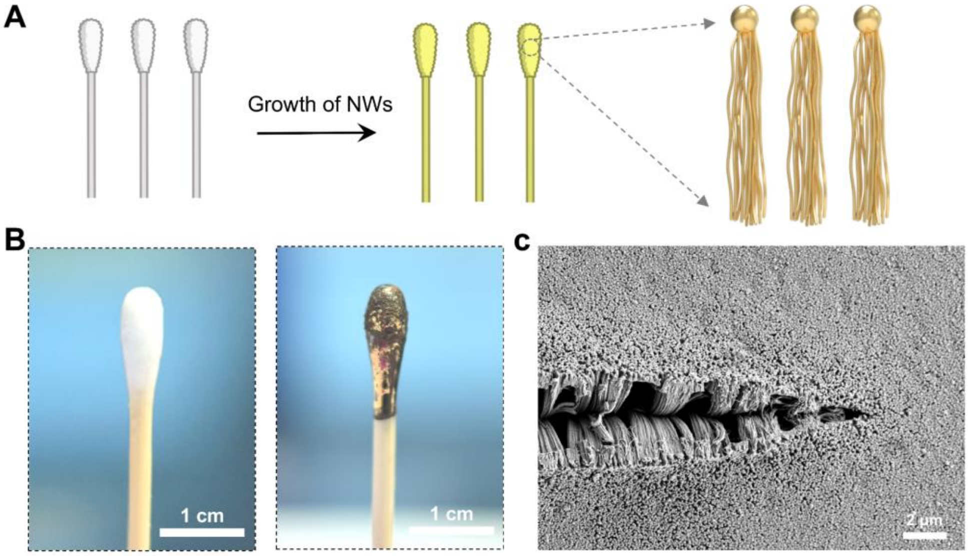

Fig. S2. VA Au–Ag NWs on medical cotton swabs. (A) Schematic illustration of the growth of VA Au–Ag NWs on the surface of the medical cotton swabs. (B) The pictures of medical cotton swabs before and after the VA Au-Ag NWs growth. (C) SEM image showing the successful formation of VA Au–Ag NWs on the medical cotton swabs. Image created with Microsoft PowerPoint, 3ds Max, and BioRender. Chen, Y. (2026) https://BioRender.com/inbqo7j.

1.3 HRTEM Characterization of the VA Au–Ag NWs

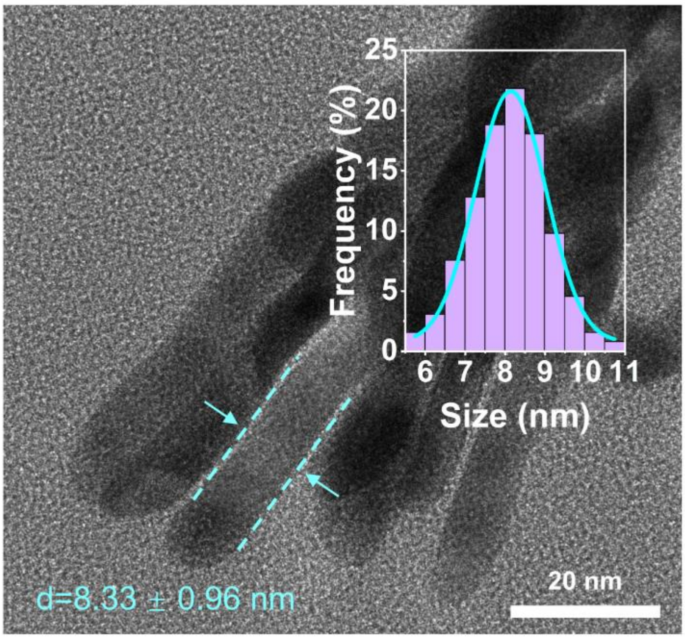

Fig. S3. HRTEM image of the VA Au–Ag NWs. The average diameter is measured to be 8.33 ± 0.96 nm, as indicated by the dashed lines and arrows. The inset shows the corresponding size distribution histogram of the nanowire diameters, fitted with a Gaussian function.

1.4 XRD Pattern of the VA Au–Ag NWs

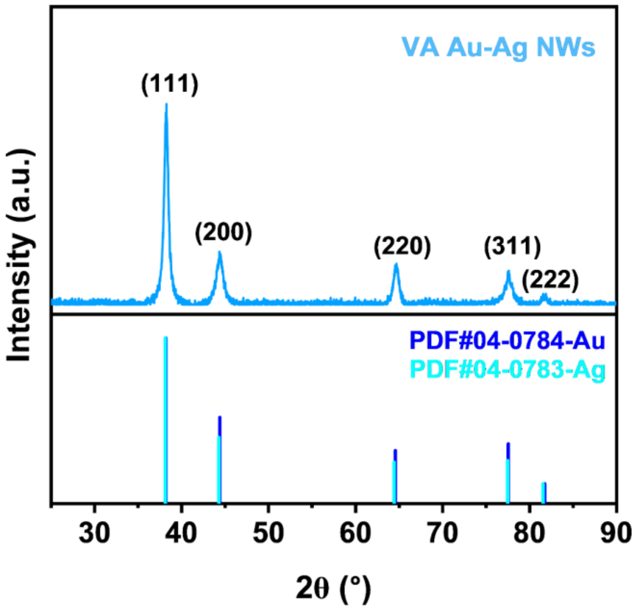

Fig. S4. XRD pattern of the VA Au–Ag NWs. Peaks are compared with the standard substance plot (PDF card) of Au and Ag.

1.5 EDS Characterization of the VA Au–Ag NWs

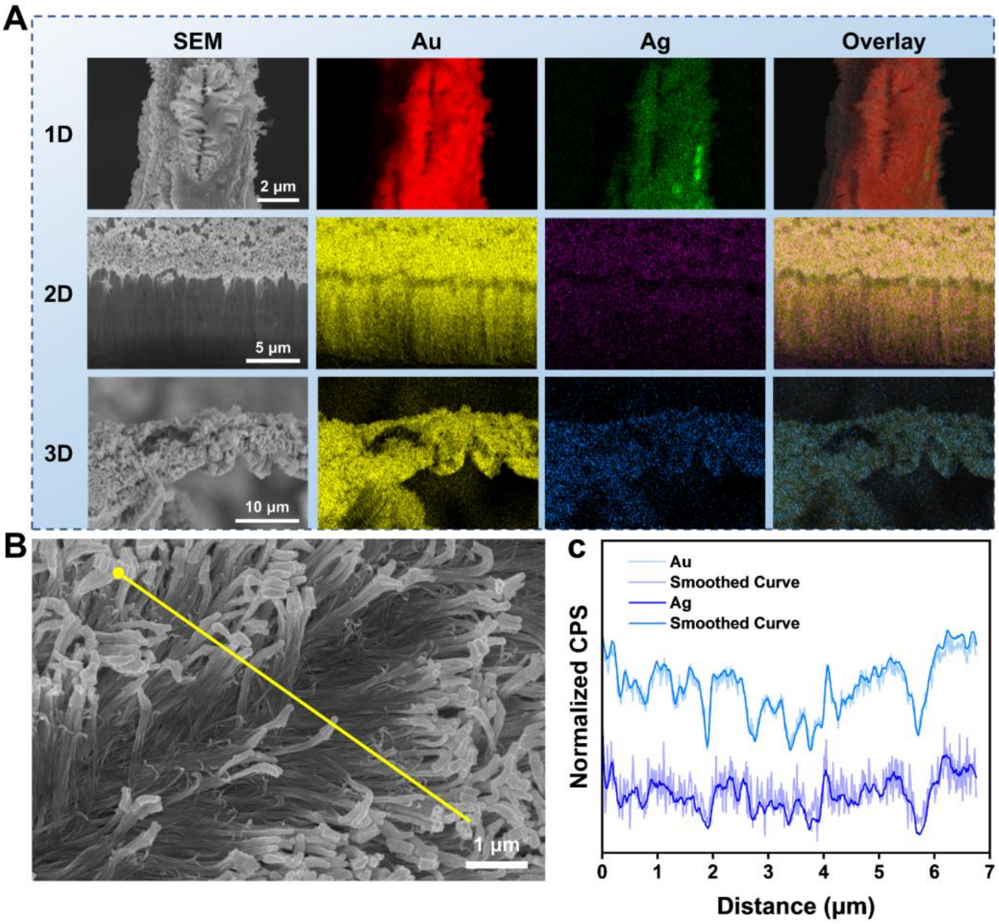

Fig. S5. Morphology and elemental distribution of VA Au–Ag NWs on 1D-3D substrates. (A) EDS elemental mapping images (HAADF, Au, Ag, overlay) of VA Au-Ag NWs growing on 1D~3D substrates. (B) SEM image of the VA Au–Ag NWs, with the yellow line indicating the line-scan path. (C) Corresponding EDS line-scan profiles along the yellow path in (B), showing the distribution of Au (blue) and Ag (purple).

1.6 Formation Mechanism of VA Au–Ag NWs

To further validate the proposed formation mechanism of VA Au–Ag NWs, we conducted a series of control experiments by replacing AgNPs with AuNPs and gold nanostars (AuNSs) as seeds. In these experiments, AgNPs were added only during the final growth stage. As shown in **fig. S6**, vertical NWs growth was still observed under both conditions. Simultaneously, EDS analysis confirmed that the resulting nanowires were composed of both Au and Ag, further supporting the proposed growth mechanism of VA Au–Ag NWs.

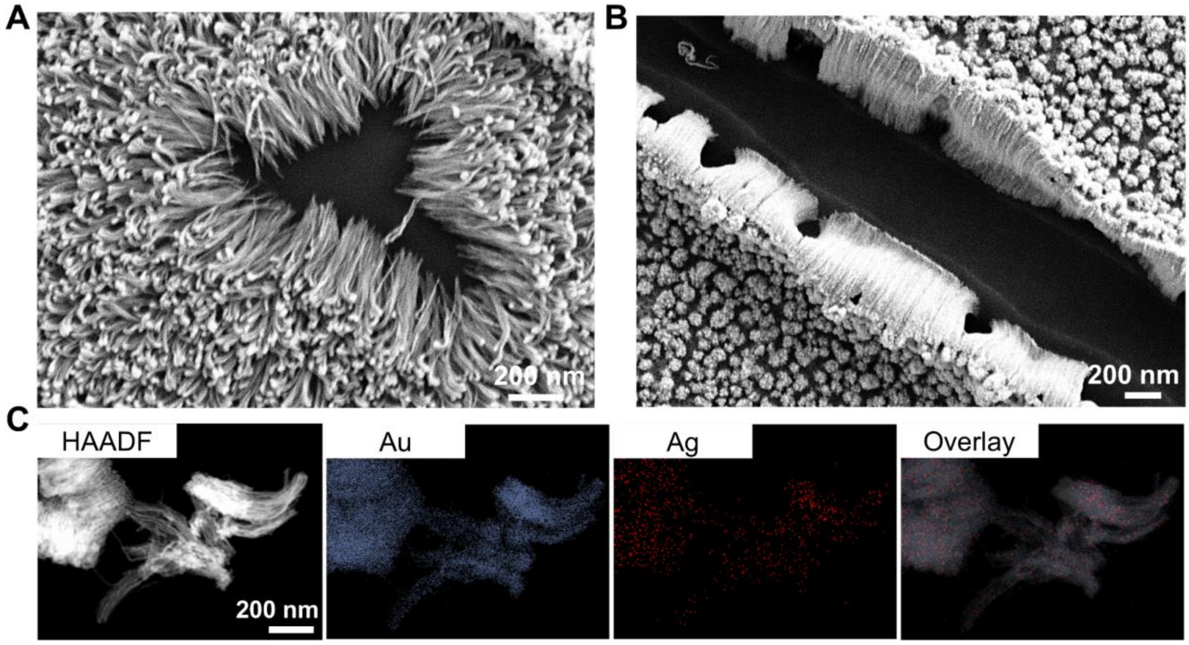

Fig. S6. Morphology and elemental characterization of vertical NWs seeded by AuNPs and AuNSs. (A and B) SEM images of vertical NWs seeded by AuNPs and AuNSs, respectively. (C) EDS elemental mapping images.

Section 2. SERS Performance of 2D VA Au–Ag NWs FlexoSERS Films

2.1 SERS sensitivity

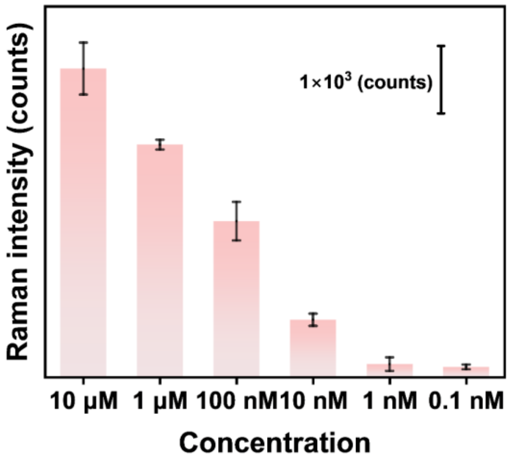

Fig. S7. Raman intensities of the typical peaks (~1078 cm^{-1}) at different R6G concentrations.

2.2 Hydrophobicity

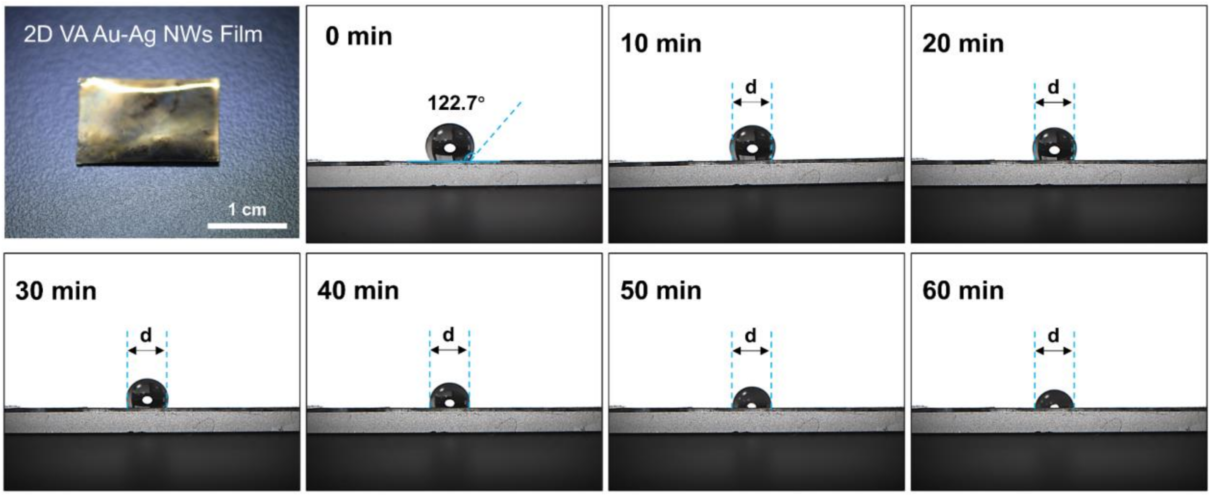

Fig. S8. Water contact angle measurement of the 2D VA Au–Ag NWs FlexoSERS film over a period of 1 hour. The first image shows a photograph of the actual 2D VA Au–Ag NWs FlexoSERS film.

2.3 Optical Micrographs of 2D VA Au-Ag NWs FlexoSERS Films with Varying Seed Concentrations

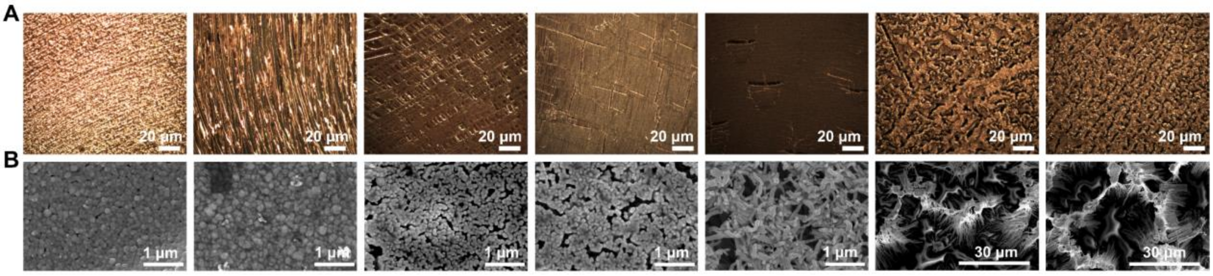

Fig. S9. 2D VA Au–Ag NWs FlexoSERS films grown under different seed (AgNP) concentrations. (A) Optical microscopy images and (B) SEM images of the resulting nanowire films. From left to right, the seed concentrations are 1000 × 10^{-7} M, 500 × 10^{-7} M, 250 × 10^{-7} M, 125 × 10^{-7} M, 62.5 × 10^{-7} M, 31.25 × 10^{-7} M, and 15.625 × 10^{-7} M, respectively.

2.4 SERS Spectra of 2D VA Au-Ag NWs FlexoSERS Films with Varying Seed Concentrations

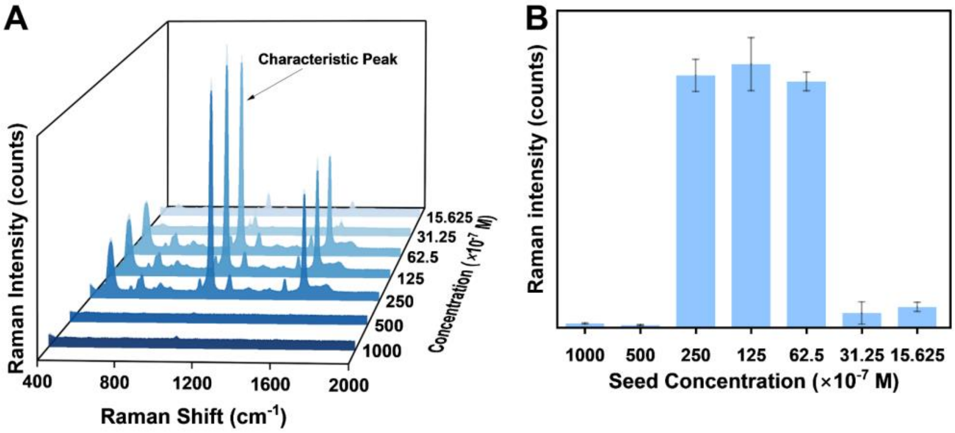

Fig. S10. SERS spectra of 2D VA Au-Ag NWs FlexoSERS films with varying seed (AgNP) concentrations. (A) SERS spectra with different AgNP seed concentrations. (B) Raman intensities of the typical peaks (~1078 cm^{-1}) at different seed concentrations.

2.5 FEM Simulation

To further elucidate the seed concentration–dependent SERS response of VA Au–Ag NWs observed in **fig. S10**, finite element method (FEM) simulations were performed based on a nanosphere array model. The results revealed that the electromagnetic hotspots initially intensified and subsequently diminished as the interparticle spacing increased (**fig. S11**), which accounts for the trend in SERS signal variation shown in **fig. S10**.

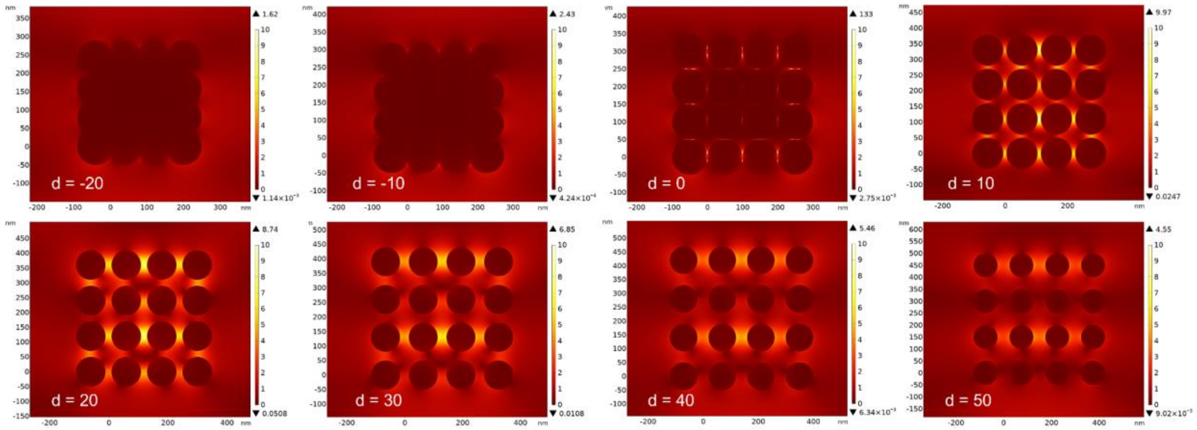

Fig. S11. Local EM field distribution around the nanosphere array model with varying array spacing.

2.6 SERS Stability

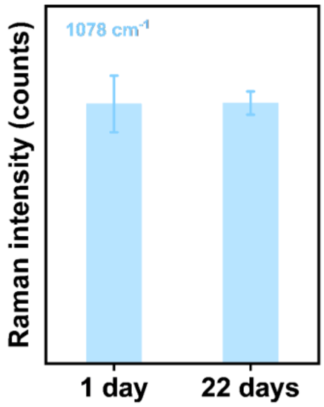

Fig. S12. Raman intensities of the characteristic peak at ~1078 cm^{-1} on day 1 and day 22.

2.7 SERS pH Sensing Mechanism

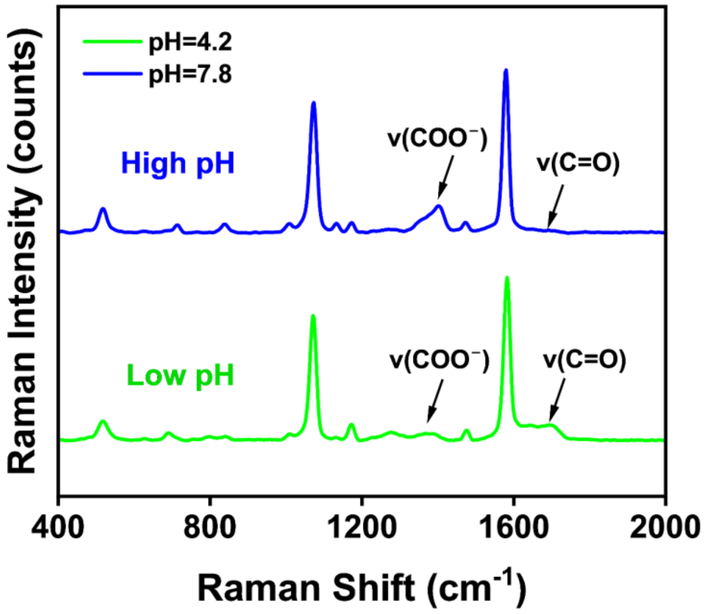

Fig. S13. SERS spectra of VA Au-Ag NWs FlexoSERS film in McIlvaine buffer at high and low pH.

2.8 Calibration Curve

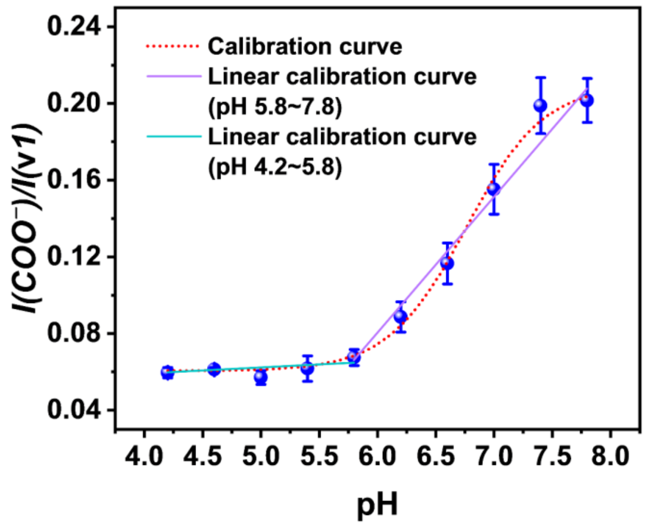

Fig. S14. Calibration curve for the relative intensity of the *ν*COO⁻ to the benzene ring ν1 mode against pH.

2.9 Mechanical Durability

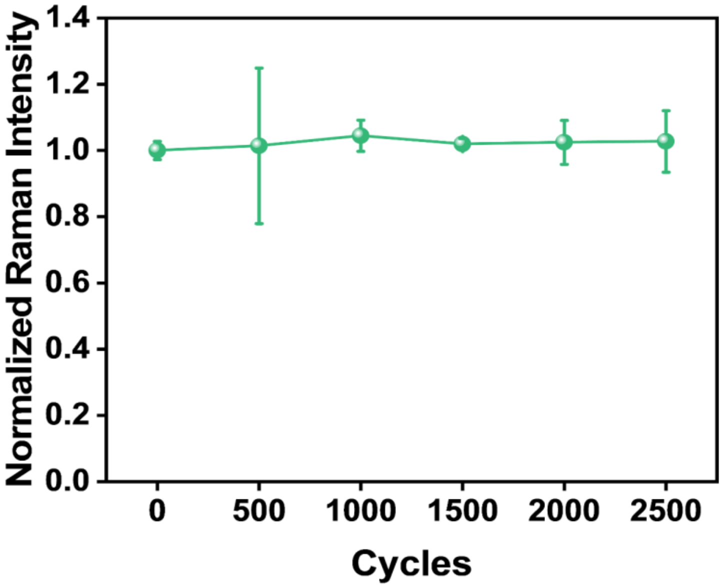

Fig. S15. Raman intensity retention of the 2D VA Au-Ag NWs FlexoSERS film at different cycles that extracted from Fig. 4f.

Section 3. Biocompatibility of VA Au-Ag NWs

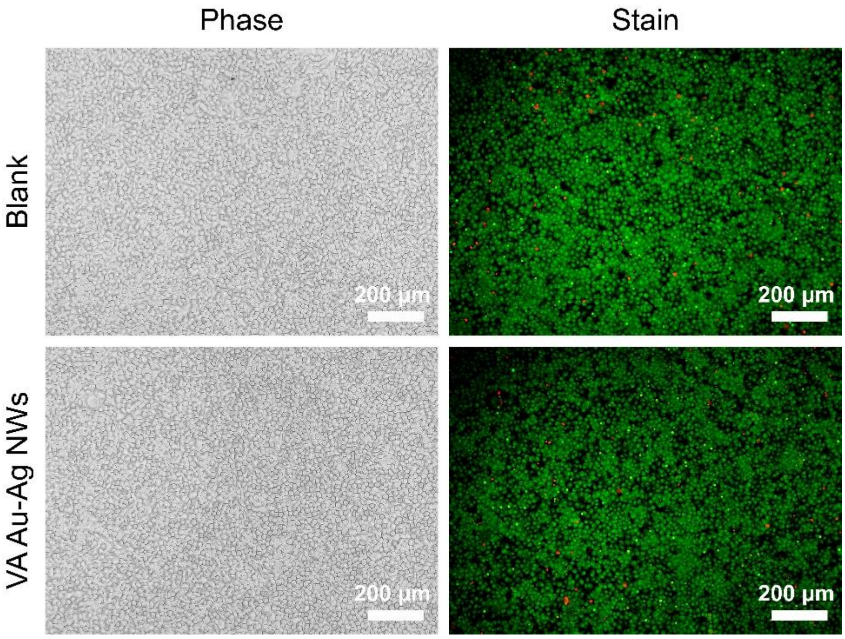

Fig. S16. Cells stained by AO&EB.

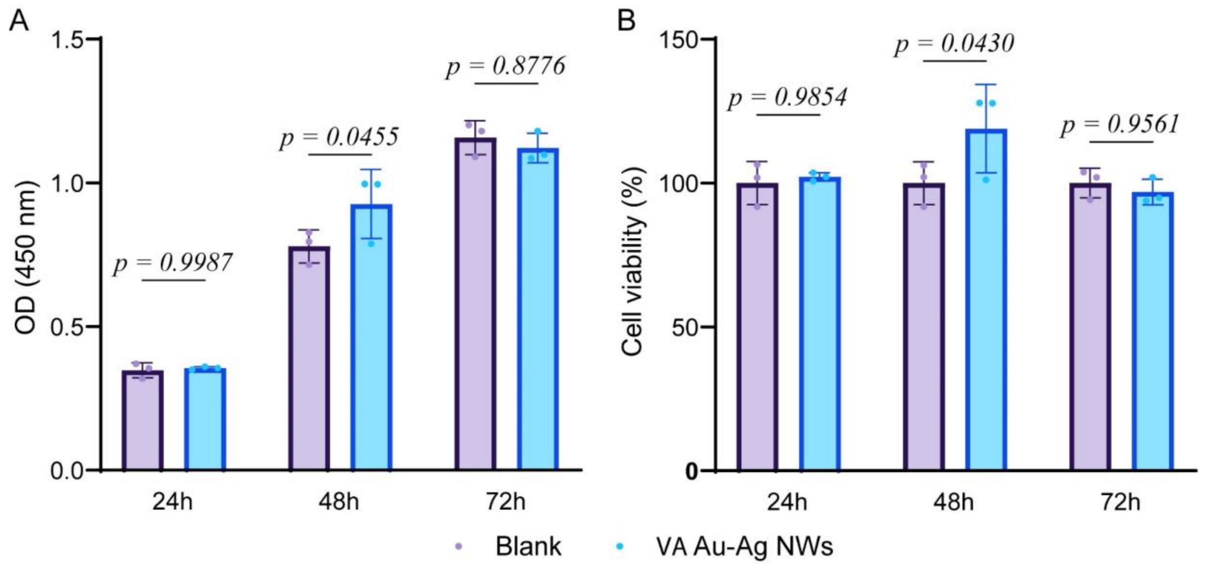

Fig. S17. Cytocompatibility evaluation of VA Au–Ag NWs by CCK-8 assay. (A) Optical density (OD) value. (B) Cell viability, detected by the CCK-8 assay. All values are mean ± s.d. (n = 3). All statistical significance is labeled.

Section 4. 3D Sponge/NWs Elastronic Electrode for Electrophysiological Sensing

4.1 Twisting Test

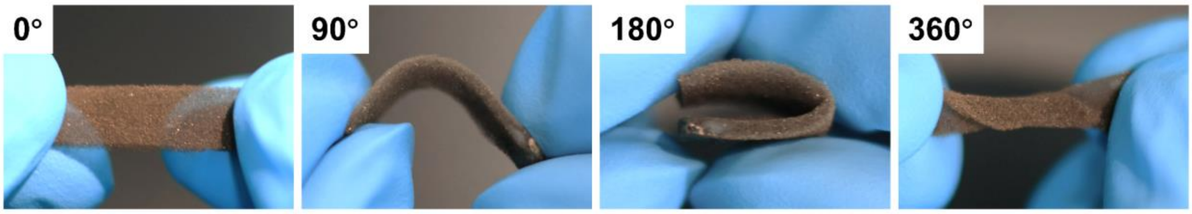

Fig. S18. Pictures of 3D sponge/NWs elastronic electrode withstanding twisting (0°~360°) without destruction.

4.2 Comparison of Skin–Electrode Impedance

We compared the skin–electrode impedance between 3D sponge/NWs elastronic electrodes and commercial gel electrodes (**fig. S18A**). To ensure a fair comparison, the commercial hydrogel was carefully removed from the commercial electrode housing, and the 3D sponge/NWs elastronic electrode was mounted in its place for skin contact (**fig. S18B**). The skin-electrode impedance of the 3D sponge/NWs dry electrode was measured to be 183.97 kΩ at 100 Hz, slightly higher than that of a commercial gel electrode (65.16 kΩ). Nevertheless, both values are sufficiently low to ensure reliable electrophysiological signal acquisition, as supported by prior studies(*30, 49*).

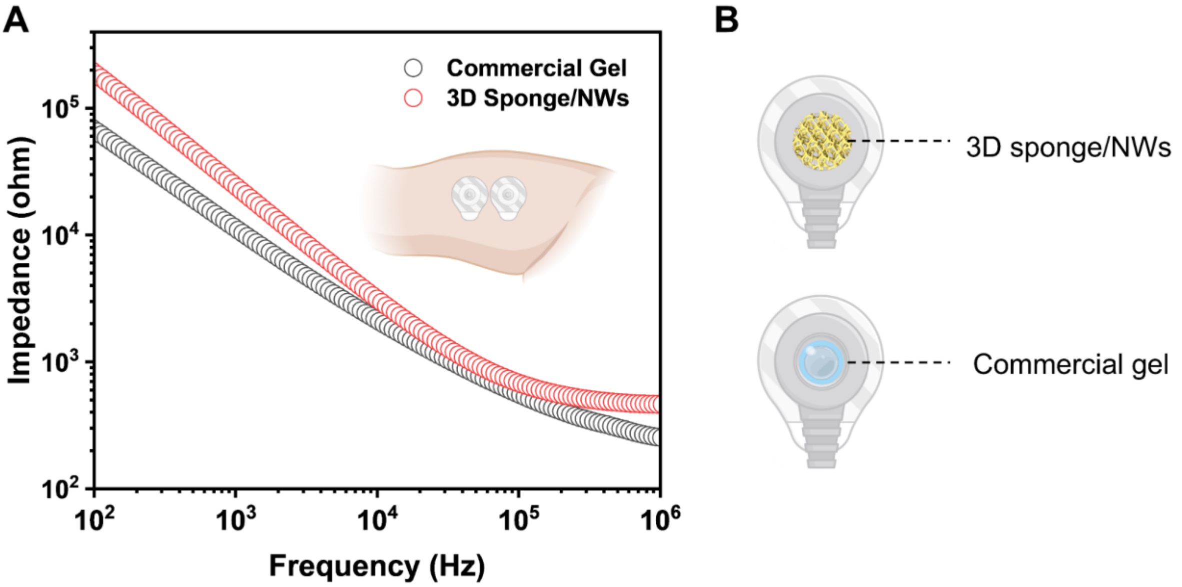

Fig. S19. Comparison of skin-electrode impedance. (A) Comparison of skin–electrode impedance between 3D sponge/NWs elastronic electrodes and commercial gel electrodes. The insert is a schematic diagram of electrode placement. (B) Structural schematics of the 3D sponge/NWs elastronic electrode (top) and the original commercial gel electrode (bottom). Images created with Microsoft PowerPoint, Blender, and BioRender. Chen, Y. (2026) https://BioRender.com/inbqo7j.

4.3 ECG Signal Recordings during Walking

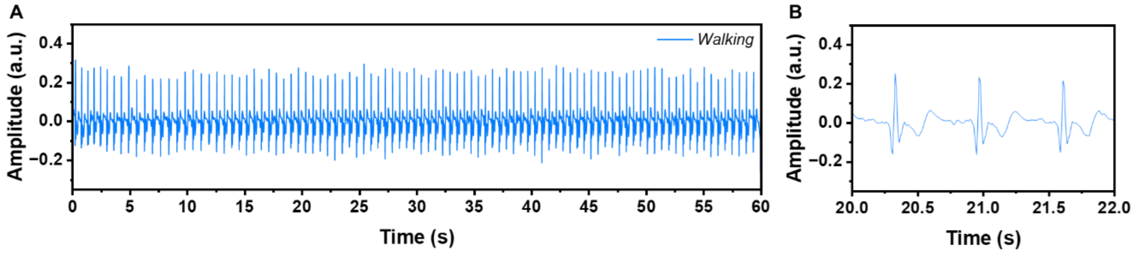

Fig. S20. ECG signal recordings during walking. (A) ECG signal recordings during walking. (B) Magnified signal segments.

4.4 ECG Signal Recordings under Sweating

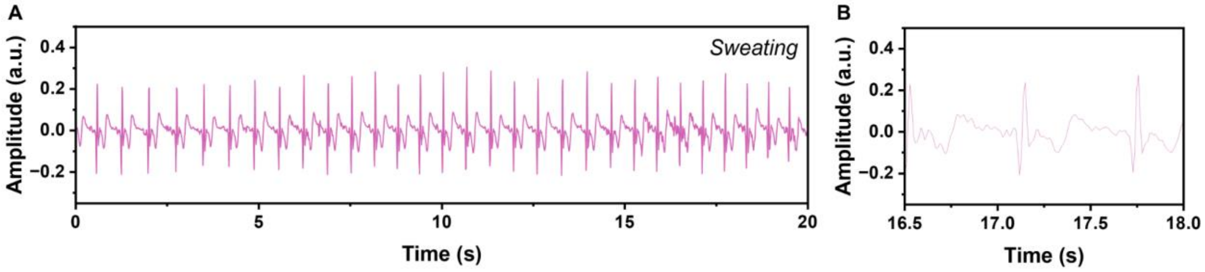

Fig. S21. ECG signal recordings under sweating. (A) ECG signal recordings after 10 minutes of vigorous activity. (B) Magnified signal segments.

4.5 Long-Term Electrophysiological Monitoring

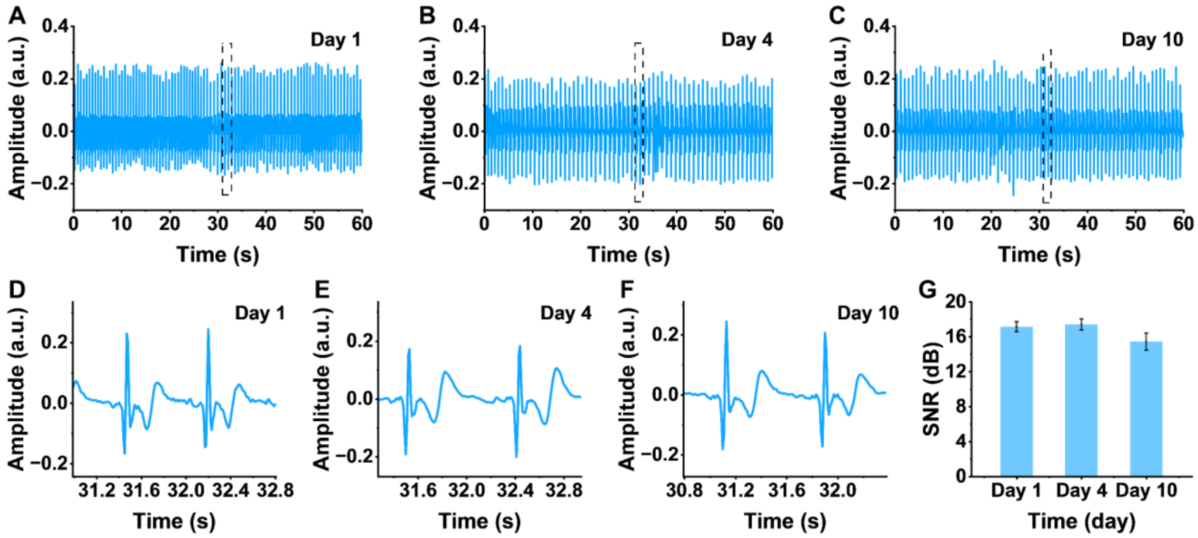

Fig. S22. Long-term electrophysiological monitoring. (A-C) ECG signals collected on days 1 (A), 4 (B), and 10 (C). (D-F) Magnified signal segments. (g) SNR on days 1, 4, and 10.

4.6 Bi-LSTM Network for Sleep–Wake State Classification

To accurately distinguish between sleep and wake states, a neural network architecture was constructed, as illustrated in **fig. S23A**. Specifically, the collected raw data were segmented based on a predefined time window, resulting in a series of data segments. These segments were then divided into training and testing sets at a 7:3 ratio. The training data were fed into a bidirectional long short-term memory (Bi-LSTM) network, which generated two sequences of features. These sequences were concatenated along the feature dimension and subsequently passed through a dropout layer to enhance learning capacity and reduce the risk of overfitting. The resulting features were then processed by a fully connected layer followed by a Softmax activation layer to produce the classification output, where '0' indicates sleep and '1' indicates wakefulness. The predicted results were compared with the ground-truth labels to compute the loss, which was then backpropagated to optimize the network parameters.

Furthermore, classification performance under different time window settings was assessed by analyzing the accuracy, precision, recall, and F1-score, calculated as follows:

$$Acc = \frac{TP + TN}{N}, P = \frac{TP}{TP + FP}, R = \frac{TP}{TP + FN}, F1 = \frac{2 \times P \times R}{P + R}$$

Here, N denotes the total number of samples, while TP, TN, FP, and FN represent true positives, true negatives, false positives, and false negatives, respectively (**fig. S23B-E**). The results indicate that when the time window is set to 3, the four performance metrics exhibit satisfactory values. However, as the length of the time window increases, the classification performance tends to deteriorate and becomes less stable. This degradation is likely caused by the inclusion of incidental or sporadic events, such as involuntary movements that occur during the night, within longer time windows. These events may introduce noise and interfere with accurate state classification. Therefore, a time window of 3 s was selected in this study to ensure a prediction accuracy exceeding 97%.

The trained network was then used to predict the labels of the testing segments, and the outcomes were compared with the actual labels to generate a confusion matrix, where only 210 instances were incorrectly classified, as shown in **fig. S24A**. The classification outcome is also visualized in **fig. S24B**, which is consistent with **Fig. 5J**. To evaluate robustness, the network was independently tested 10 times under the same time window setting, yielding a consistent

prediction accuracy of 98.7% with a relative standard deviation (RSD) of 0.11%, indicating strong performance and reliability (**Fig.e S24C**).

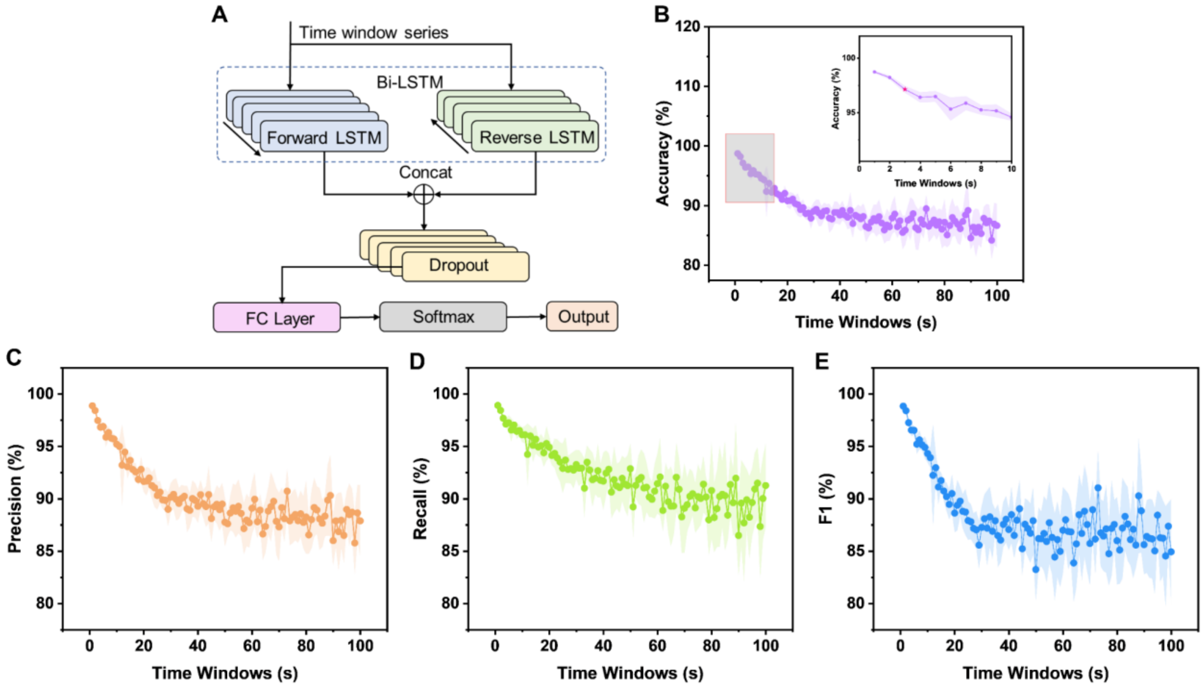

Fig. S23. Performance evaluation of the Bi-LSTM-based neural network. (A) Schematic illustration of the Bi-LSTM-based neural network. (B) Accuracy as a function of time window length. (C-E) Precision (C), Recall (D), and F1-score (E) plotted as a function of time window duration, all showing a gradual decrease with longer time windows.

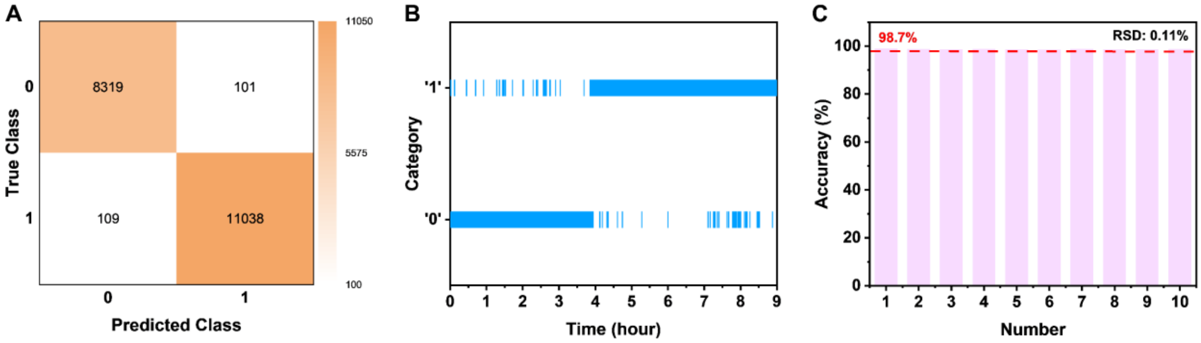

Fig. S24. Classification performance of the Bi-LSTM model for long-term monitoring. (A) Confusion matrix corresponding to a time window of 3s. (B) Binary classification results over a 9-hour continuous recording session, where '1' denotes wakefulness and '0' denotes sleep. (C) Prediction accuracy across 10 independent tests under the same time window of 3s.

4.7 Comparison Between Commercial Gel Electrodes and 3D Sponge/NWs Elastronic Electrodes

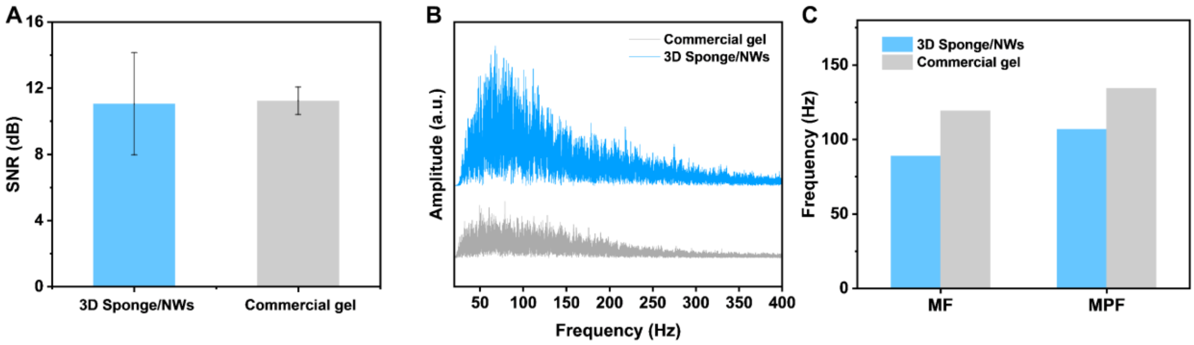

Fig. S25. Comparison of electrophysiological signal features during fist clenching. The signal-to-noise ratio (A), frequency spectrum (B) and their mean power frequency (MPF) and mean frequency (MF) (C) recorded by the 3D sponge/NWs elastronic electrode and commercial gel electrode during fist clenching.

4.8 sEMG Signals Recorded During Finger Movements

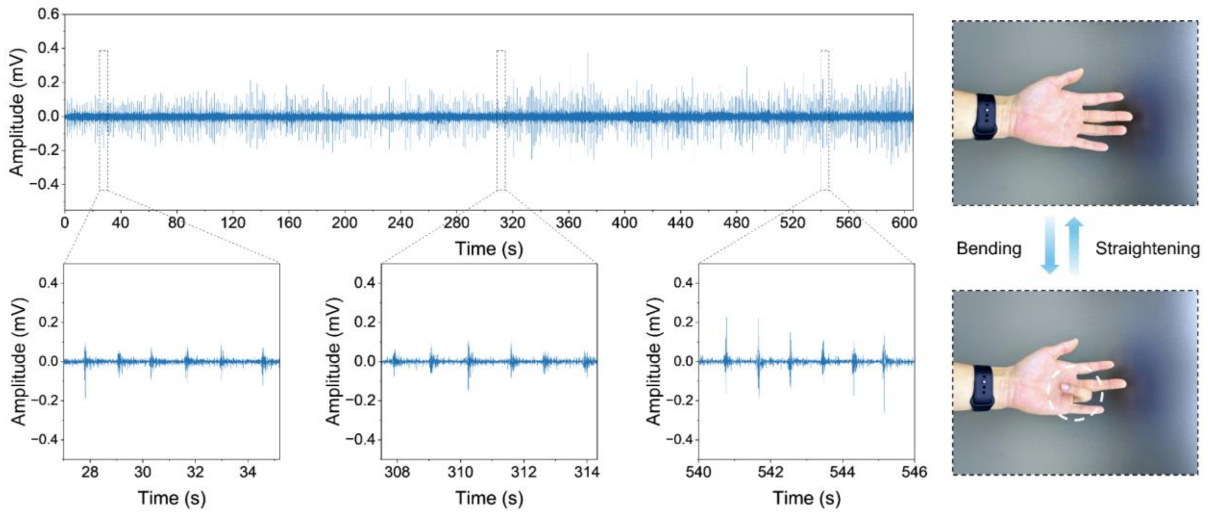

Fig. S26. sEMG signals recorded during ring finger movements over the 600-second monitoring period. The right panel shows images of the finger during flexion and extension. Magnified signal segments corresponding to flexion and extension phases are displayed below.

4.9 sEMG Signals Monitoring During Keyboard Typing and Mouse Clicking

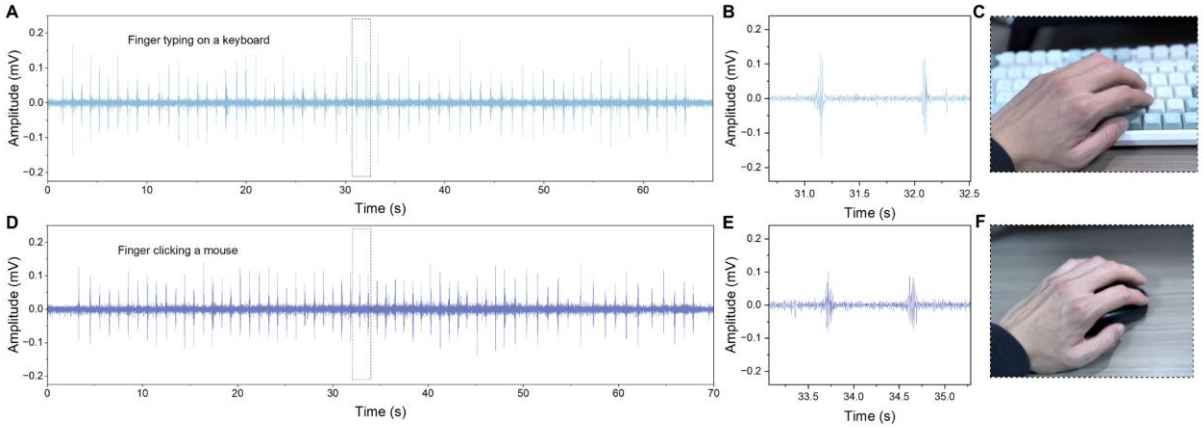

Fig. S27. sEMG signals monitoring during keyboard typing and mouse clicking. (A) sEMG signals recorded during finger typing on a keyboard. (B) Magnified signal segments corresponding to keyboard typing. (C) Picture of keyboard typing. (D) sEMG signals recorded during mouse clicking. (E) Magnified signal segments corresponding to mouse clicking. (F) Picture of mouse clicking.
